# Dynamic Molecular Cocrystals with Alkyl Chain Dependent Thermosalient Phase Transitions

**DOI:** 10.1002/advs.202502692

**Published:** 2025-04-15

**Authors:** Jiantao Meng, Yuan Su, Hang Zhu, Jie Zhang, Ting Cai

**Affiliations:** ^1^ Department of Pharmaceutics School of Pharmacy China Pharmaceutical University Nanjing 211198 P. R. China; ^2^ Department of Pharmaceutical Engineering School of Engineering China Pharmaceutical University Nanjing 211198 P. R. China

**Keywords:** alkyl chain, cocrystal engineering, dynamic molecular crystals, polymorphic phase transition, thermosalient

## Abstract

Thermally responsive molecular crystals exhibiting programmable mechanical motions hold significant promise for applications in smart actuators, sensors, and drug delivery systems. However, achieving precise control over their phase transition thermodynamics remains a fundamental challenge. A series of isomorphic 5‐fluorocytosine/fatty acid cocrystals is reported where the phase transition temperatures vary across an interval of 100 K with increasing alkyl chain. Two distinct transition pathways are unveiled: i) a cooperative single‐crystal‐to‐single‐crystal transition (II‐III) accompanied by explosive mechanical motions, and ii) a reconstructive transition (I‐III) following classical nucleation‐growth mechanisms. The cooperative phase transition (II‐III) induces remarkable expansion, with a striking +64.4% expansion along the layer stacking direction and a −16.9% contraction perpendicular to the (001) plane, leading to dynamic phenomena such as jumping, rotating, and splitting. Notably, the transition temperatures (*T*_t, II‐III_) exhibit linear dependence on coformer chain length (from C10 to C18), a correlation attributed to interlayer hydrophobic interactions. This work provides a versatile approach for designing molecular crystals with tunable thermo‐mechanical properties, offering new opportunities for advanced applications in dynamic functional materials.

## Graphical abstract

A series of cocrystals containing 5‐fluorocytosine and fatty acids with varying alkyl chain lengths are synthesized, demonstrating a systematic modulation of thermosalient phase transition temperatures across a broad range (≈100 K). This study reveals a linear correlation between interlayer hydrophobic interactions and transition temperatures, offering a strategic approach for designing dynamic materials with tunable thermal‐responsive properties.

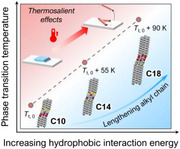

## 1 Introduction

Dynamic molecular crystals, a class of materials that undergo self‐actuation and locomotion in response to external stimuli such as mechanical stress, heat, light, magnetic, or electric field, are gaining attention across chemistry, materials science, and engineering.^[^ ^1^ ^]^ Their ability to rapidly and efficiently transduce energy makes them ideal candidates for dynamic devices such as actuators, sensors, robotics, and energy harvesting systems.^[^ ^2^ ^]^ Among these, salient crystals, or “jumping crystals”, stand out due to their unique ability to directly convert internal or external energy into mechanical work with high energy efficiency and rapid response times.^[^ ^3^ ^]^ Based on the type of stimulus, salient crystals are further classified as thermosalient, photosalient, or mechanosalient system.^[^ ^4^ ^]^ The themosalient effect is typically driven by a cooperative phase transition, or martensitic transition, in response to temperature changes, involving rapid and anisotropic changes in the unit cell.^[^ ^5^ ^]^ These structural transformations generate significant strain within the crystal lattice, and its sudden release results in macroscopic mechanical motion such as jumping, rolling, rotating, flipping, and even splitting.^[^ ^6^ ^]^ In comparison, photosalient crystals exhibit similar mechanical effects upon exposure to light, where photochemical or photothermal processes induce lattice strain, leading to motion.^[^ ^4b,c^ ^]^ Similarly, mechanosalient crystals respond to mechanical stimuli, such as pressure or force, and undergo abrupt shape changes or fractures due to stress‐induced phase transitions.^[^ ^4d^ ^]^ While thermosalient and photosalient crystals are primarily used in thermal and optical systems, mechanosalient crystals find applications where mechanical forces are involved, such as in pressure sensors and force‐responsive actuators. To date, over 50 thermosalient crystals have been identified, many discovered serendipitously.^[^ ^7^ ^]^ Despite considerable progress, the design of molecular crystals with tunable thermal‐mechanical responses, such as adjustable response temperatures and dynamic behaviors, remains a challenge.

A common approach to designing thermosalient materials involves modulating known thermosalient compounds through halogen or isotopic substitution and variations in side chains.^[^ ^8^ ^]^ For instance, halogenated Schiff bases show thermomechanical responses that are sensitive to halogen type, and variations in alkyl chain length in naphthalene dimide derivatives significantly influences the temperature at which thermosalient transitions occur.^[^ ^9^ ^]^ However, these methods can lead to unpredictable results due to complex changes in crystal packing and phase transition mechanisms.^[^ ^10^ ^]^ Cocrystallization, a versatile approach in crystal engineering, has emerged as a promising strategy for tuning various physical properties, including solubility, melting points, and mechanical behaviors.^[^ ^11^ ^]^ By forming supramolecular structures through noncovalent interactions, involving hydrogen bonding, halogen bonding, π–π interactions, and van der Waals forces, cocrystallization provides a facile means of designing novel crystals.^[^ ^12^ ^]^ The relatively weak yet tunable intermolecular interactions in cocrystals facilitate reversible phase transitions and dynamic mechanical responses, making this strategy particularly attractive for designing thermosalient materials with controllable properties. Although several cocrystals with thermosalient effects have been reported recently,^[^ ^13^ ^]^ the potential of cocrystallization in the rational design of dynamic molecular crystals remains underexplored.

**Figure 1:**
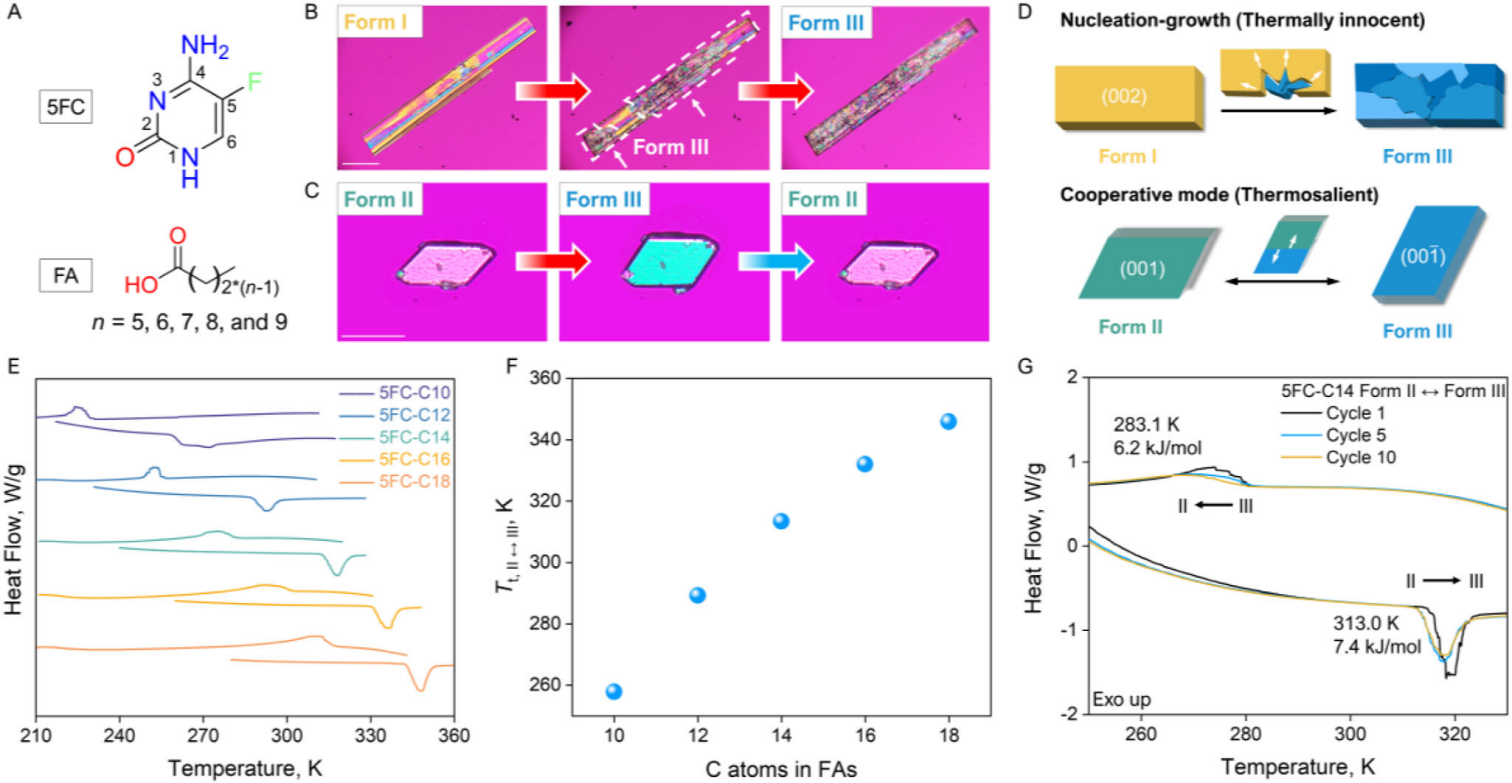
Polymorphism and thermal behaviors of 5FC‐C2*n* cocrystals. A) Molecular structure of 5‐fluorocytosine (5FC) and fatty acids (FA). B, C) Optical microscopic images showing different crystal habits of the polymorphs of the 5FC‐C14 cocrystals: long‐plate form I and parallelogram‐like form II or III. Two different phase transition behaviors were observed in this system. Scale bar: 100 µm. B) Nucleation‐growth phase transition from form I to form III at 370 K. C) Single‐crystal‐to‐single‐crystal (SCSC) phase transition from form II to form III. D) Schematic diagram illustrating the polymorphic transformation in 5FC‐C2*n* cocrystals. E–G) Thermal profiles of phase transitions in 5FC‐C2*n* cocrystals, monitored by DSC with a 10 K min^−1^ heating/cooling rate. E) DSC curves of powder samples of 5FC‐C2*n* cocrystal form II. All cocrystals exhibit a reversible transition. F) Temperature variation of the form II to form III transition in 5FC‐C2*n* cocrystals as a function of fatty acid chain length. G) Effects of thermal cycling on the thermosalient phase transitions (II‐III) in crystals of 5FC‐C14.

Alkyl chain engineering, which involves systematically modifying the length or bulkiness of alkyl side chains within molecular structures, is widely employed in materials science to regulate phase transitions, mechanical flexibility, solubility, and electronic properties.^[^ ^14^ ^]^ The incorporation of alkyl chains enhances structural flexibility, often leading to conformational polymorphism and facilitating cooperative transition through order‐disorder rotational motions.^[^ ^15^ ^]^ Additionally, the thermal vibration of dynamic alkyl side chains can induce colossal thermal expansion, further influencing phase transition behavior.^[^ ^16^ ^]^ In this work, we integrate cocrystallization with alkyl chain engineering to construct thermally responsive crystals and systematically modulate their phase transitions. A series of fatty acid cocrystals were synthesized using 5‐fluorocytosine (5FC), leveraging the robust carboxyl‐amino and carboxyl‐pyridine supramolecular synthons.^[^ ^17^ ^]^ The resulting isomorphous 5FC cocrystals with fatty acids (C2*n*, *n* = 5–9) (**Figure** **1**A) exhibit two distinct polymorphic phase transition mechanisms: a cooperative phase transition (II‐III) with thermosalient behaviors (jumping, rotating, and splitting), and a nucleation‐growth type transition (I‐III) without thermosalient effects. The thermosalient transition temperature (*T*_t, II‐III_) was highly sensitive to alkyl chain length, varying by ≈100 K from C10 to C18, while the nucleation‐growth transition (I–III) remained unaffected. The thermosalient transition involves a reorientation of molecular layers, resulting a striking expansion along the layer stacking direction. A linear correlation was established between the thermosalient transition temperature (*T*_t, II–III_) and the strength of interlayer hydrophobic interactions. This revealed that systematic modulation of alkyl chain length allows precise control of interlayer interactions and cooperative phase transition temperatures, enabling the modulation of thermosalient behavior across a wide temperature range. To the best of our knowledge, this is the first report to establish a quantitative correlation between weak interlayer interactions and phase transition temperatures, as well as the first demonstration of a tunable transition temperature spanning such an extensive range within a single family of cocrystals. These findings offer a new strategy for designing responsive materials with tunable thermo‐mechanical properties and advance the development of dynamic molecular crystals via cocrystallization.

## 2 Results and Discussion

### 2.1 Preparation of 5FC‐ C2*n* Cocrystals

We synthesized a series of 5‐fluorocytosine (5FC) cocrystals (Figure 1A), designated as 5FC‐C2*n* (*n* = 5–9), by dissolving equimolar 5FC and the respective fatty acids, and subsequently recrystallizing them at either ambient temperature or 340 K from mixed organic solvents (see details in the Experimental Section). Trimorphic forms were found in these 5FC‐C2*n* cocrystals and were characterized by powder X‐ray diffraction (PXRD), Raman spectroscopy, and differential scanning calorimetry (DSC) (Figures S1–S5, Supporting Information). Specifically, monoclinic form I crystallized as long plates and was observed in the 5FC‐C14, 5FC‐C16, and 5FC‐C18 systems (Figure 1B; Figure S3, Supporting Information). Triclinic form II or form III, as described in further detail below, which crystallized as parallelograms, was observed across all 5FC‐C2*n* systems (Figure 1C; Figure S3, Supporting Information). PXRD patterns indicated that these cocrystals share isostructural features within the same polymorph (Figures S1 and S2, Supporting Information).

### 2.2 Phase Transitions of 5FC‐C2*n* Cocrystals

The DSC analysis revealed distinct thermal behaviors for the polymorphic forms. Form I melted directly upon heating at 20 K min^−1^, while the transition from form I to form III occurred at slower heating rates (Figure S4, Supporting Information). As determined using melting point and enthalpy of fusion, the transition temperatures (*T*_t, I‐III_) remained nearly constant across the 5FC‐C14, 5FC‐C16, and 5FC‐C18 systems (Table S1, Supporting Information). In contrast, form II underwent a first‐order endothermic transition (II to III) before melt (Figure S5, Supporting Information). The II‐III phase transition was reversible during heating and cooling cycles (Figure 1E). Interestingly, the transition temperature (*T*_t, II‐III_) was highly sensitive to alkyl chain length, with *T*_t, II‐III_ spanning ≈100 K (250 to 350 K) as chain length increased from C10 to C18 (Figure 1F). Moreover, single‐crystal DSC revealed a “sawtooth” transition peak profile, indicative of thermosalient effects (Figure 1G; Figure S6, Supporting Information). In subsequent thermal cycles, the transition temperatures (*T*_t, II‐III_) remained nearly constant, while the sawtooth‐like transition peaks gradually smoothed out. Though the modulation of cooperative crystal transition temperatures through molecular design and crystal engineering has been explored, systematic modulation in transition temperature across such an extensive range has not been reported.

Hot stage microscope was used to investigate thermal events in the 5FC‐C2*n* cocrystals. Face indexing results revealed that the basal face exposed to the hot stage is parallel to the (002) plane of form I, $\left(00\bar{1}\right)$ plane of form II and the (001) plane of form III, respectively (Figures S7 and S8, Supporting Information). Distinct transition behaviors were observed, reflecting differences in the underlying mechanisms for the I‐III and II‐III transitions. For the I‐III transition, a nucleation‐growth mechanism was evident. Heating 5FC‐C14 form I initiated the phase transition at several points within the crystal, progressing slowly without a defined direction (Figure 1B; Figures S9 and S10, Supporting Information). Complete transition into form III polycrystals required over 15 min at 370 K. This nucleation‐growth behavior was consistent across 5FC‐C16 and 5FC‐C18 systems (Figure S10, Supporting Information). In contrast, the II‐III phase transition followed a cooperative mechanism. 5FC‐C14 form II underwent a rapid, single‐crystal‐to‐single‐crystal transition characterized by swift changes in birefringence (Figure 1C; Figure S11 and Movies S1 and S2, Supporting Information). The morphology and birefringence of the crystal were fully restored upon cooling, confirming the reversibility of this transformation. Importantly, at temperatures near *T*_t, II‐III_, crystal movement was observed during both heating and cooling cycles (Figures S12 and S13, Supporting Information), as detailed in the following section.

### 2.3 Thermosalient Effects During Reversible Forms II to III Phase Transition

**Figure 2:**
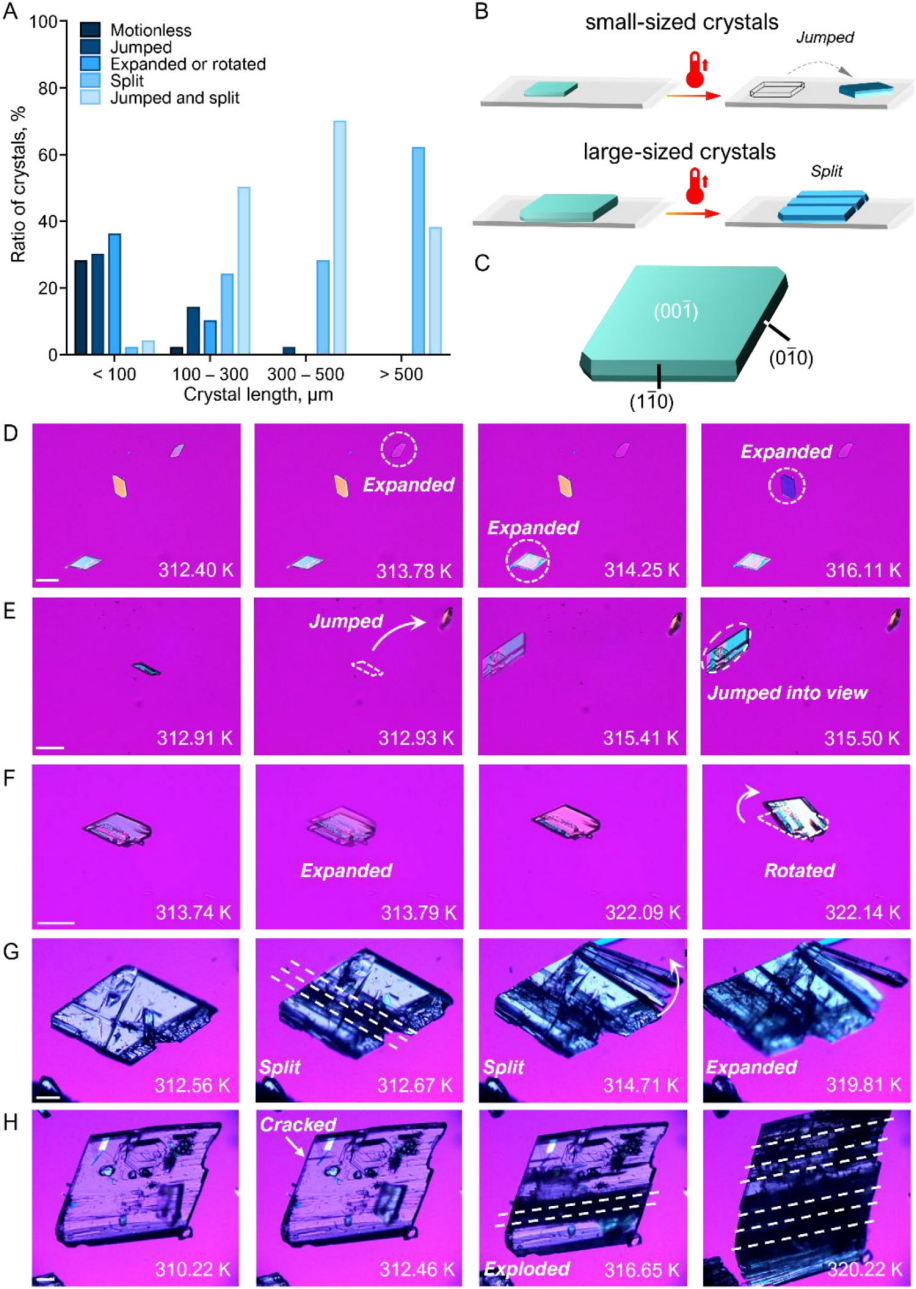
Thermosalient phase transitions in 5FC‐C14 cocrystals. A) The distribution of the relative ratio of motions in 5FC‐C14 cocrystals during the II‐III transition. A total of 50 crystals in each size range were selected for statistical analysis. Some smaller‐sized crystals exhibit no morphological changes other than birefringence variations during heating‐cooling cycles, which is defined as motionless. B) Schematic representation of the thermomechanical behaviors observed in crystals of different sizes during the II‐III phase transition. C) Crystal morphology of form II with indexed face. D–G) In situ monitoring of the thermosalient effects during the heating of form II on the (001) face at a heating rate of 20 K min^−1^ on an open hot stage. Scale bar: 100 µm. D) Several crystals immersed in silicone oil expanded, and their birefringence changed accordingly (Movie S2, Supporting Information). E) A small crystal jumped to the edge of view, followed by a larger crystal that jumped into the field of view (Movie S3, Supporting Information). F) A crystal expanded and displaced first, then rotated (Movie S4, Supporting Information). G, H) A larger crystal underwent intense, explosive motion during phase transition (Movies S5 and S6, Supporting Information). White dash lines represent the parallel cleavage planes in the crystal.

To investigate the kinematic details of the thermosalient effect, 5FC‐C14 cocrystals were selected as a representative system. A total of 50 individual 5FC‐C14 crystals were analyzed using a hot stage microscope at a heating rate of 20 K min^−1^. Their thermomechanical responses were categorized based on motion type and crystal size. The results indicate that the phase transition occurs extremely rapidly, and the thermomechanical responses were largely unaffected by varying heating rates (Figure S14, Supporting Information). These observations revealed a range of behaviors, including expanding, rotating, jumping, splitting, and combinations thereof (**Figure** **2**D–H). Analysis of size‐dependent behavior (Figure 2A) showed that smaller crystals typically rotated or jumped while maintaining their crystal integrity, whereas larger crystals were more likely to exhibit explosive behavior. Moreover, ≈30% of crystals with lengths <100 µm exhibited no motions but underwent significant expansion along the short axis of the $\left(00\bar{1}\right)$ surface upon heating (Figure 1C; Figure S11, Supporting Information). Interestingly, splitting crystals consistently displayed cracking along the [010] direction, accompanied by expansion (Figure 2F,G). The directional splitting and expansion behaviors suggest the anisotropy of the crystal structure which facilitates macroscopic crystal deformation along specific crystallographic directions. However, we did not observe specific orientation dependency in the jumping or rotation of the crystals.^[^ ^18^ ^]^ The size‐dependent dynamic behaviors have been observed in other molecular thermosalient crystals as well. Usually, larger crystals tend to disintegrate under stress due to structural defects (e.g., dislocations, vacancies, or grain boundaries) and surface flaws, which act as stress concentrators. In contrast, smaller crystals, with fewer defects, can better withstand stress and typically undergo elastic deformation (expansion) or displacement (jumping or rotating) instead of cracking (Figure S15, Supporting Information). Similar to 5FC‐C14 cocrystals, other cocrystals also exhibit intense thermosalient effects (Figure S16 and Movies S11–S14, Supporting Information), which is consistent with the DSC characterization.

### 2.4 Molecular Origin of the Phase Transitions and Thermosalient Effects

**Figure 3:**
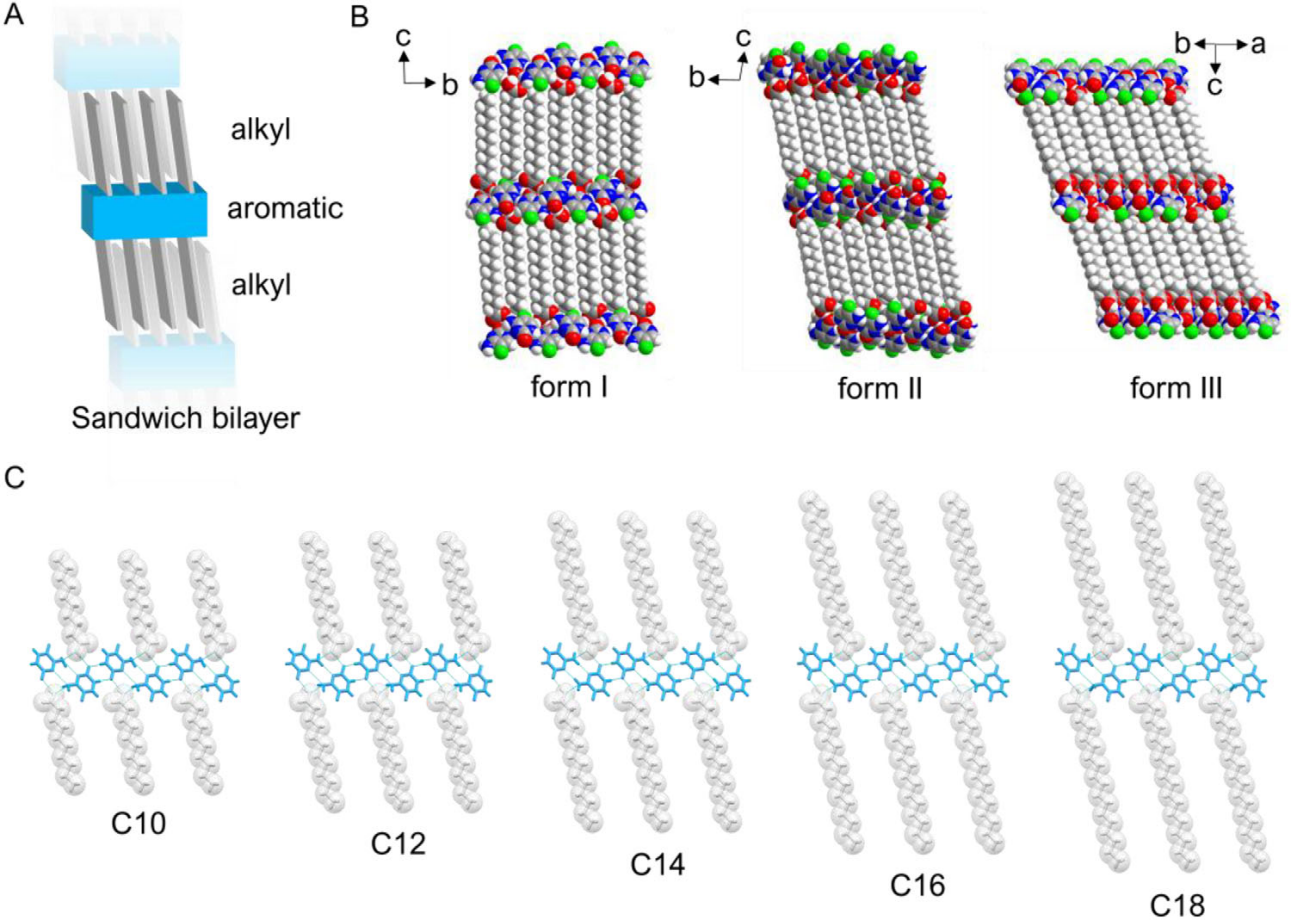
Crystal structure of the 5FC‐C2*n* polymorphs. A) Schematic representation of the segregated packing arrangement in 5FC‐C2*n* cocrystals. B) Space‐filling model of 5FC‐C14 polymorphs, showing the distinct alkyl and aromatic layers. C) Projections of the ($\bar{1}$00) plane for form II of the 5FC‐C2*n* with *n* = 5, 6, 7, 8, and 9.

**Figure 4:**
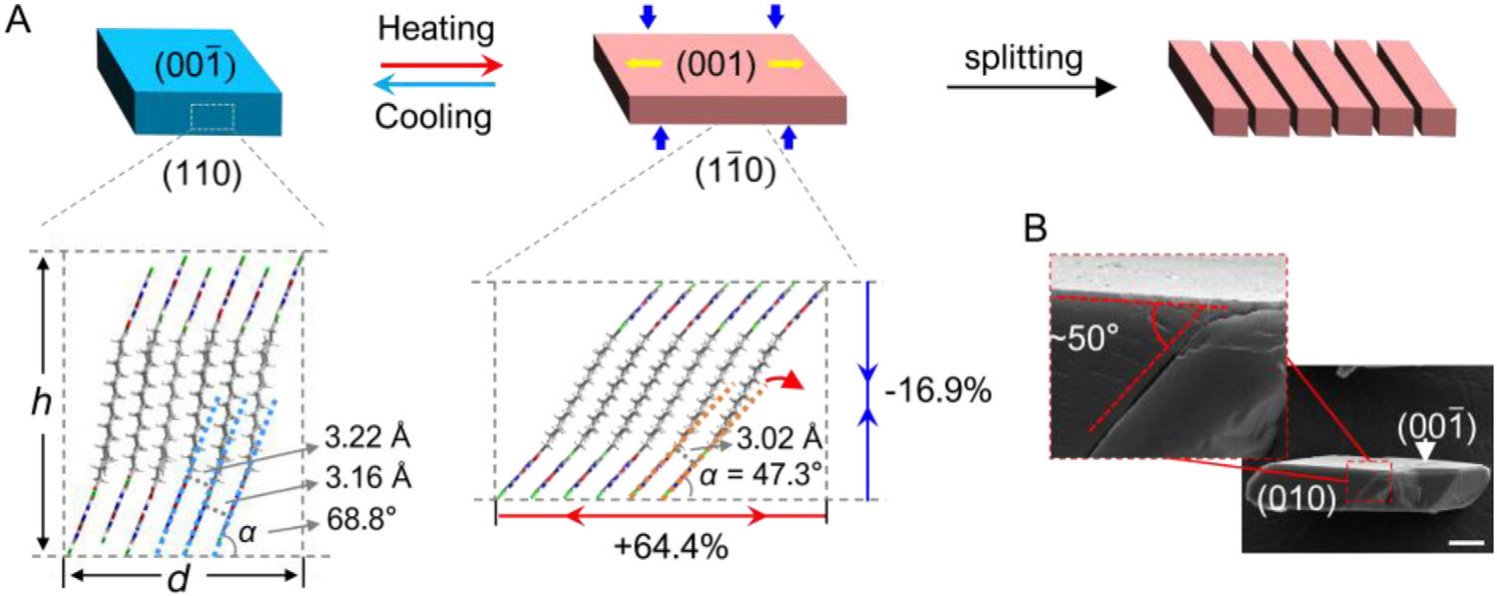
Molecular mechanism of the thermosalient effect. A) Schematic diagram of crystal expansion and splitting during heating. A perspective view of the layer packing mode of 5FC‐C14 form II at 160 K and form III at 323 K. The layer stack with their 5FC ribbons tilted at angle (given as *α* in the text) against the (001) plane. Upon heating from 160 to 323 K, the distance between the (001) plane decreases by 16.9%, while the direction along the layer stacking elongated by 64.4%. B) Scanning electron microscopy (SEM) image of a crystal of the 5FC‐C14 form III after splitting. Insert is enlarged view of the highlighted sections.

To elucidate the mechanisms underlying the phase transitions and thermomechanical behaviors of 5FC‐C2*n* cocrystals, single‐crystal X‐ray diffraction (SCXRD) was conducted, with crystallographic parameters summarized in Tables S2 and S3 (Supporting Information). In all crystal forms, fatty acids and 5FC molecules organize into distinct layered arrangements (**Figure** **3**A,B). The 5FC molecules are linked into 1D ribbons by $R_{2}^{2}\left(8\right)$ dimers through N─H∙∙∙N and N─H∙∙∙O hydrogen bonds. The fatty acid molecules interact with these aromatic ribbons via COO─H···O bonds and adopt a fully extended conformation, forming sandwich‐like alkyl bilayers (Figure S17, Supporting Information). Form I crystallizes in a monoclinic system, featuring corrugated alkyl bilayers where the aromatic ribbons are tilted relative to the alkyl layers. These bilayers stack antiparallel along the *c*‐axis, and the alkyl chains are offset along the *a*‐axis (Figure S18, Supporting Information). In contrast, forms II and III crystallize in a triclinic system and exhibit nearly flat sandwich bilayers (Figure 3C; Figure S19, Supporting Information). In these forms, adjacent alkyl bilayers are highly interdigitated along the fatty acid long axis, resulting in an extended layered structure. These molecular layers are arranged parallel to each other, with a slight tilt relative to the [001] direction. The layered arrangement is primarily stabilized by van der Waals interactions, as the closest contacts between adjacent aromatic rings are >4.0 Å (Figures S18 and S19, Supporting Information), suggesting that π‐π interaction is minimal in these forms. A key structural difference between forms II and III lies in their interlayer arrangement: form II exhibits alternating interlayer distances (e.g., 3.22 and 3.16 Å in 5FC‐C14), whereas form III demonstrates a uniform interlayer distance (3.02 Å in 5FC‐C14) (**Figure** **4**A; Figure S20, Supporting Information). This structural similarity facilitates the cooperative phase transition observed between these two forms. In contrast, the transformation of monoclinic form I to triclinic form III involves significant reconstruction of the crystal structure and proceeds via a nucleation‐to‐growth mechanism.

Crystal structure analysis revealed that form II, across varying alkyl chain lengths, is isomorphous, with the lattice parameter *c* systematically increasing as the alkyl chain length increases (Figure 3C). This suggests a consistent cooperative transition mechanism across the 5FC‐C2*n* systems. Accordingly, the 5FC‐C14 system was selected as a representative example to investigate the molecular mechanism of thermosalient phase transition (II‐III). In form II at 160 K, the molecular layers stacked along the $\left[1\bar{1}0\right]$ direction, with the aromatic ribbon tilted at an angle *α* of ≈68.8° relative to the (001) plane, which is the basal face exposed to the hot stage (Figure 4A). Upon heating from 160 to 260 K, *α* decreases slightly to 68.3° (Figure S21, Supporting Information). However, during the transition from form II to form III, the molecular layers undergo a dramatic reorientation toward the (001) plane, with *α* decreasing significantly to 47.3° (Figure 4A). This reorientation induces notable anisotropic volume changes in the crystal: a significant expansion (+64.4%) along the layer stacking directions and a contraction perpendicular to the (001) plane (−16.9%) (Figure 4A). Macroscopically, this expansion is reflected in significant shape changes of crystal, particularly the pronounced expansion of the short axis, which corresponds to the stacking direction of molecular layers (Figure 4A). The parallelogram‐shaped 5FC‐C14 cocrystal exhibits expansion (15.4% and 9.5% along opposing edges), surpassing typical uniaxial expansions in thermosalient systems.^[^ ^2e^ ^]^ Notably, the biaxial ≈10% positive expansion in 5FC‐C2*n* cocrystals suggests unique application potential compared to conventional counterparts (Figure S22, Supporting Information). In contrast, the in‐plane changes within the molecular layer are negligible (<1.5%) (Figure S23, Supporting Information). The significant expansion generates substantial strain within the crystal. This strain is often released through movement, potentially compromising crystal integrity and leading to explosive disintegration. Scanning electron microscope (SEM) analysis of the split crystals (Figure 4B) reveals that explosive splitting typically occurs along the crystal's long axis, forming an angle of ≈50°. This suggests that the crystals tend to split along a direction approximately parallel to the $\left(11\bar{5}\right)$ plane (form III) (Figure S24, Supporting Information). This plane nearly aligns with the molecular layers and is stabilized by van der Waal's interactions between these layers. Energy framework analysis further reveals that the interlayer interaction energy (73.46 kJ mol^−1^) is significantly lower than the total intralayer interaction energies (145.48 kJ mol^−1^), indicating that splitting along the $\left(11\bar{5}\right)$ under stress plane is energetically more favorable (Figure S25, Supporting Information).^[^ ^19^ ^]^ Similar expansion (+54.1% along the layer stacking directions) and uniaxial contraction (−24.1% perpendicular to the (001) plane) were also observed in 5FC‐C10 cocrystal during its II‐III phase transition, demonstrating a shared mechanism for the thermosailent behavior within this family of cocrystals (Figure S26, Supporting Information).

### 2.5 Thermosalient Phase Transition Dependence of Alkyl Chain

**Figure 5:**
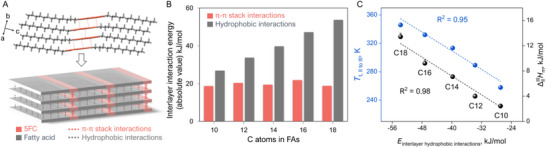
Interlayer interaction analysis. A) The layered structure and schematic diagram of 5FC‐C14 form II. 5FC/FA molecules and their corresponding interactions are shown in pink and gray, respectively. B) Changes in hydrophobic and π‐π stack interactions energies per molecule of form II with varying alkyl chain lengths. In order to simplify the calculations, interactions below 15 kJ mol^−1^ were neglected. C) The linear relationship between the transition temperature (*T*_t, II‐III_), transition enthalpy, and intralayer hydrophobic interaction energies.

In this work, we observed that the thermosalient transition in 5FC‐C2*n* cocrystals is highly sensitive to the alkyl chain length, with a tunable transition temperature spanning ≈100 K (250–350 K) as the alkyl chain length increases from C10 to C18. This variation in transition temperature offers the potential for controlling thermosalient behavior over a broad temperature range, which is crucial for practical application. Alkyl chains are flexible, and their dynamics often play a critical role in phase transition behaviors.^[^ ^9b^ ^]^ To access the impact of alkyl chain dynamics on the thermosalient transition temperature, we analyzed thermal ellipsoid plots (Figures S27–S29, Supporting Information), which offers insights into atom vibrations within the crystal lattice.^[^ ^14^, ^16^ ^]^ These plots reveal increased thermal motion of fatty acid chains at elevated temperatures, with noticeable disorder occuring near transition temperature. However, structural analysis shows that, despite these increased motions, only minor conformational changes occur during the phase transition, as evidenced by the molecular overlap in forms II and III (Figure S30, Supporting Information). Furthermore, the phase transition temperatures do not correlate with the average atomic displacement parameters (*U*_eq_), indicating that alkyl chain dynamics play a minimal role in the phase transition (Figure S31 and Table S4, Supporting Information). As discussed above, the phase transition from form II to form III is primarily driven by the cooperative reorientation of molecular layers in response to the temperature changes. While alkyl chain disorder and thermal vibrations are present, they have minimal impact on the transition. Therefore, interlayer interactions are the key factors determining the thermal stability of these cocrystals. Analysis of intermolecular interaction energies using CrystalExplorer shows that the total energy of intermolecular interactions, including π‐π stacking and alkyl chain−alkyl chain interactions, is dependent on the alkyl chain length (**Figure** **5**A,B). Interactions between the aromatic ribbons (π‐π stack) are primarily van der Waals interactions, as evidence by the long centroid‐to‐centroid distance and minimal overlap between the aromatic rings (Figure S25, Supporting Information) and these interactions remain nearly constant across all the 5FC‐C2*n* system (Figure 5B). In contrast, the chain−chain interactions are mainly ascribed to hydrophobic interactions (or attractive dispersion forces) between the alkyl chains. Although individual dispersion forces are weak, their cumulative effect becomes significant as the alkyl chain length increases, leading to substantial interlayer interaction.^[^ ^14e^ ^]^ We observed that chain−chain interactions, especially the interlayer part, systematically increase with increasing alkyl chain length. Furthermore, a linear relationship between the energy of these hydrophobic interactions and the thermosalient transition temperature (*T*_t, II‐III_) was established (Figure 5C). Based on these findings, we conclude that the dependence of the thermosalient transition temperature on alkyl chain length is primarily governed by the hydrophobic interaction within the crystal structure, highlighting the critical role of weak interaction in dictating thermo‐mechanical responsiveness. While the influence of intermolecular interaction on thermosalient behavior has been recognized previously,^[^ ^8^, ^10^, ^20^ ^]^ this study provides the first quantitative correlation between intermolecular interaction energy and transition temperature, paving the way for the design of thermo‐mechanical responsive crystal with precise control. Additionally, an increase in melting points and a decrease in solubility were observed with the increasing alkyl chain length, attributed to enhanced hydrophobic interactions (Figures S32 and S33, Supporting Information). In 5FC‐C2*n* cocrystals, molecules with long alkyl chains often exhibit chain disorder. However, for phase transition mechanisms not dominated by molecular conformational changes, the increase in chain length and molecular disorder has a negligible impact on the phase transition behavior.^[^ ^14^, ^21^ ^]^

## 3 Conclusion

In this study, we successfully synthesized a series of 5‐fluorocytosine cocrystals with fatty acids, demonstrating the great potential of cocrystal engineering in modulating thermosalient effects. By systematically adjusting the alkyl chain length of fatty acid, the thermosalient transition temperatures (*T*_t, II‐III_) vary across an unprecedented range of 100 K. The thermosalient phase transition from form II to III is driven by molecular layer reorientations, resulting in significant expansion and dynamic mechanical motions such as jumping, rotating, and splitting. These behaviors are governed by interlayer hydrophobic interactions, which intensify with longer alkyl chains, thereby stabilizing the crystal structure and modulating the thermosalient response. For the first time, we establish a linear correlation between intermolecular interactions and thermosalient transition temperatures, providing a robust framework for designing dynamic materials with tunable thermal properties. This cocrystallization‐based strategy offers significant potential for applications in smart materials, temperature‐responsive devices, and other fields requiring precise control over thermal behaviors.

## 4 Experimental Section

### Materials and Reagents

5‐Fluorocytosine (5FC) (purity: 98%, form I), n‐caproic acid (C6) (purity: 99%), n‐caprylic acid (C8) (purity: 99%), n‐decanoic acid (C10) (purity: 99%), lauric acid (C12) (purity: 98%), myristic acid (C14) (purity: 99%), and palmitic acid (C16) (purity: 97%) were purchased from Aladdin Reagent Co., Ltd. (Shanghai, China). Stearic acid (C18) (purity: 98%) was purchased from Macklin Biochemical Co., Ltd (Shanghai, China). All materials were used as received.

### Preparation of 5FC‐C2*n* Cocrystal

Cocrystal form I was prepared by the slow solvent evaporation method. A solution of 6.4545 g 5FC (0.05 mol) and an equimolar amount of fatty acids (C14, C16, and C18) was added to 1 L acetonitrile‐methanol (1/3, v/v) mixed solvent and heated to 320 K until the components were completely dissolved. Single crystals were obtained by allowing the solvent to evaporate slowly at room temperature. Powder samples were prepared by grinding the single crystals. 5FC‐C12 form I can be prepared through a slurry method in an ice water bath, 5FC‐C10 form I have not been prepared yet.

Powders of form II/III were prepared using a slurry method. To a round‐bottom flask, 1.2909 g 5FC (0.01 mol) and an equimolar amount of fatty acids (C10, C12, C14, C16, and C18) were added to 50 mL acetonitrile, and the slurry was stirred at 350 K for 1 h. After cooling to room temperature, the products were filtered and dried in a vacuum oven for 12 h. Starting with a 3.5 g input of the compositions (5FC + C14), ≈3 g of the cocrystal product was obtained, yielding an actual production rate of ≈85%. Single crystals of 5FC‐C10/C12/C14/C16/C18 form II/III were obtained by slow evaporation from a 5FC and fatty acid (equimolar ratio) solution in acetonitrile‐water (5/1, v/v) mixed solvent at 340 K. Single crystals of 5FC‐C6/C8 were prepared by slow evaporation from a 5FC and fatty acid (Mole ratio, 1:100) solution in acetonitrile‐water (5/1, v/v) mixed solvent at room temperature.

### Raman Spectroscopy

Raman spectra were recorded using a Thermo Fisher DXR instrument equipped with a 532 nm externally stabilized diode laser. The samples were scanned over the range of 1800–50 cm^−1^ at a laser power of 10 mW. The data were analyzed using OMNIC software.

### Powder X‐Ray Diffraction (PXRD)

Powder X‐ray diffraction patterns were collected using a Bruker D8 Advanced diffractometer with Cu‐Kα radiation. Scans were performed in the 2*θ* range of 3–40 ° with a step size of 0.02° and step time of 0.30 s. The voltage and current applied were 40 kV and 40 mA, respectively.

### Differential Scanning Calorimetry (DSC)

DSC measurements were conducted with a TA Instrument Q2000 unit under 50 mL min^−1^ nitrogen gas purge. Accurately weighed samples (5–10 mg) were placed in sealed aluminum pans and heated from 200 K to the desired temperature at heating/cooling rate of 1, 5, 10, and 20 K min^−1^.

### Themosalient Effects Analysis

Themosalient behavior was studied using a hot stage (THMS 600, Linkam Scientific) assisted polarizing microscope (BX 53, Olympus). Crystal morphologies were observed at a heating/cooling rate of 20 K min^−1^.

### Single‐Crystal X‐Ray Diffraction (SCXRD)

Single‐crystal X‐ray diffraction data were collected using a Bruker D8 Venture X‐ray diffractometer with the Mo‐Kα radiation (λ = 0.71073 Å). Data collection, refinement of cell parameters, and simplification were performed using Bruker Saint software. Single crystal structure was determined using ShelXS‐2014/7 (Sheldrick, 2014). Absorption corrections were applied using the SADABS program, and refinement was carried out with full‐matrix least‐squares methods.

CCDC 2410432–2410440, 2413835 contain the supplementary crystallographic data for this paper. These data can be obtained free of charge from The Cambridge Crystallographic Data Centre via www.ccdc.cam.ac.uk/data_request/cif.

### Energy Framework Calculation

Energy frameworks were calculated using the crystal explorer software. The total intermolecular interaction energy with all molecules in an asymmetry unit is calculated using the B3LYP‐D2/6‐31G(d,p) electrostatic potential model, is the sum of electrostatic, polarization, dispersion, and exchange‐repulsion components with scaling factors of 1.057, 0.740, 0.871, and 0.618, respectively. The interaction energies of a selected molecule with all molecules having any atom within 3.8 Å were calculated. The interaction energies below certain energy threshold (15 kJ mol^−1^) were omitted for clarity.

### High‐Performance Liquid Chromatography (HPLC) Analysis

The content of 5FC was determined by HPLC using a Shimadzu LC‐20AT HPLC system with a C18 column (4.6 × 150 mm, 5 µm). The UV detection was performed at 265 nm, with the column maintained at 35 °C, respectively. The mobile phase consists of a methanol/phosphoric acid solution (pH = 3.5, 5/95, v/v), with a flow rate of 1.0 mL min^−1^.

### Determination of Equilibrium Solubility

Equilibrium solubility was determined using the shake‐flask method. An excess amount of cocrystals were added to 20 mL of solution with pH 6.8 and shaken at 37 °C and 100 rpm for 10 days. The samples were filtered through 0.45 µm nylon filters, diluted, and analyzed by HPLC (*n* = 3).

## Conflict of Interest

The authors declare no conflict of interest.

## Author Contributions

J.M. and Y.S. contributed equally to this work as co‐first authors. J.M., Y.S., and T.C. conceived the study, analyzed the experiment data, wrote and edited the manuscript. T.C. served as the principal investigator for the study, had full access to all of the data in the study, and took responsibility for the integrity and the accuracy of the data analysis. J.M., H.Z., and J.Z. designed and performed the experiment. J.M., Y.S., and J.Z. carried out the crystal structure analysis. All the authors discussed the results and approved the final version of the manuscript.

## Supporting information



Supporting Information

Supplemental Movie 1

Supplemental Movie 2

Supplemental Movie 3

Supplemental Movie 4

Supplemental Movie 5

Supplemental Movie 6

Supplemental Movie 7

Supplemental Movie 8

Supplemental Movie 9

Supplemental Movie 10

Supplemental Movie 11

Supplemental Movie 12

Supplemental Movie 13

Supplemental Movie 14

Supplemental Movie 15

Supplemental Movie 16

## Data Availability

The data that support the findings of this study are available from the corresponding author upon reasonable request.
