# Supplementary material for: Dynamic Molecular Cocrystals with Alkyl Chain Dependent Thermosalient Phase Transitions
Source: Adv Sci (Weinh). 2025 Apr 15;12(26):2502692. doi: 10.1002/advs.202502692 (PMC12245039; doi:10.1002/advs.202502692)
Supplement: Supplementary file 1 — Supporting Information [file ADVS-12-2502692-s017.pdf]

ADVANCED SCIENCE
Open Access

Supporting Information

for *Adv. Sci.*, DOI 10.1002/advs.202502692

Dynamic Molecular Cocrystals with Alkyl Chain Dependent Thermosalient Phase Transitions

*Jiantao Meng, Yuan Su, Hang Zhu, Jie Zhang and Ting Cai**

Supporting Information

Dynamic Molecular Cocrystals with Alkyl Chain Dependent Thermosalient Phase Transitions

Jiantao Meng,†[1] Yuan Su,†[2] Hang Zhu,[1] Jie Zhang,[1] and Ting Cai[1, 2]*

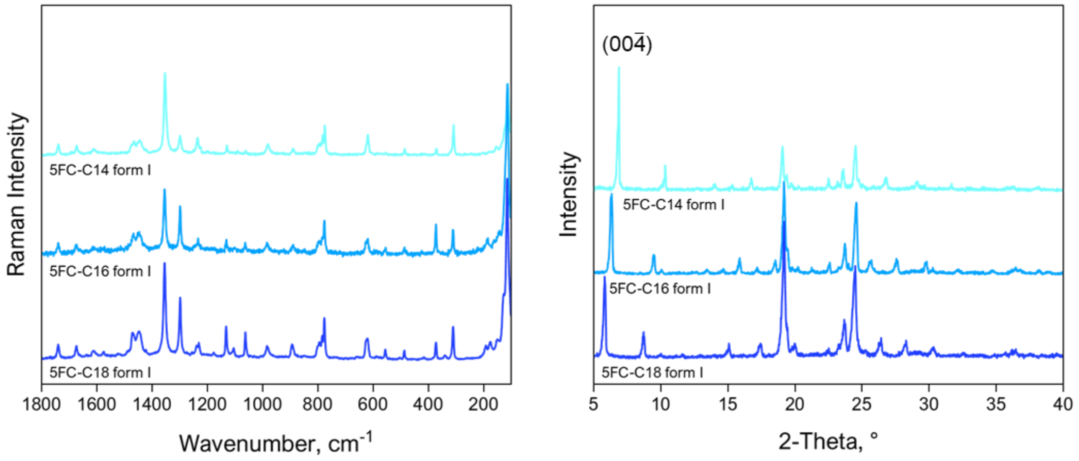

Figure S1. Raman spectra and PXRD patterns of form I in 5FC-C14, 5FC-C16 and 5FC-C18 collected at room temperature. The PXRD patterns reveal a shift of the (00$\bar{4}$) peaks to lower angles as the alkyl chain length increases.

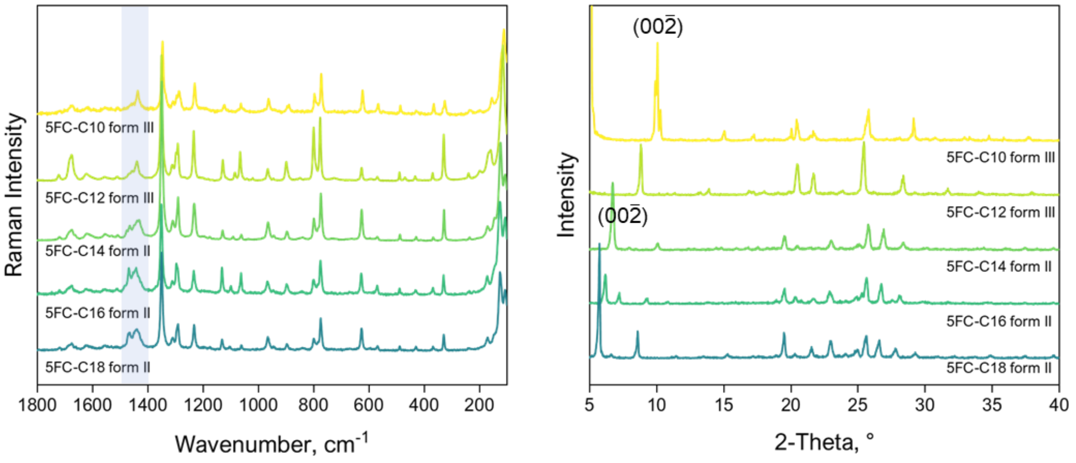

Figure S2. Raman spectra and PXRD patterns of form II or III in 5FC-C2*n* cocrystals collected at room temperature. At this temperature, 5FC-C10 and 5FC-C12 crystallize in form III, while 5FC-C14, 5FC-C16, and 5FC-C18 are in form II. Raman spectra of forms II and III show a distinct difference in the 1500-1400 cm^{-1} region (marked in blue).

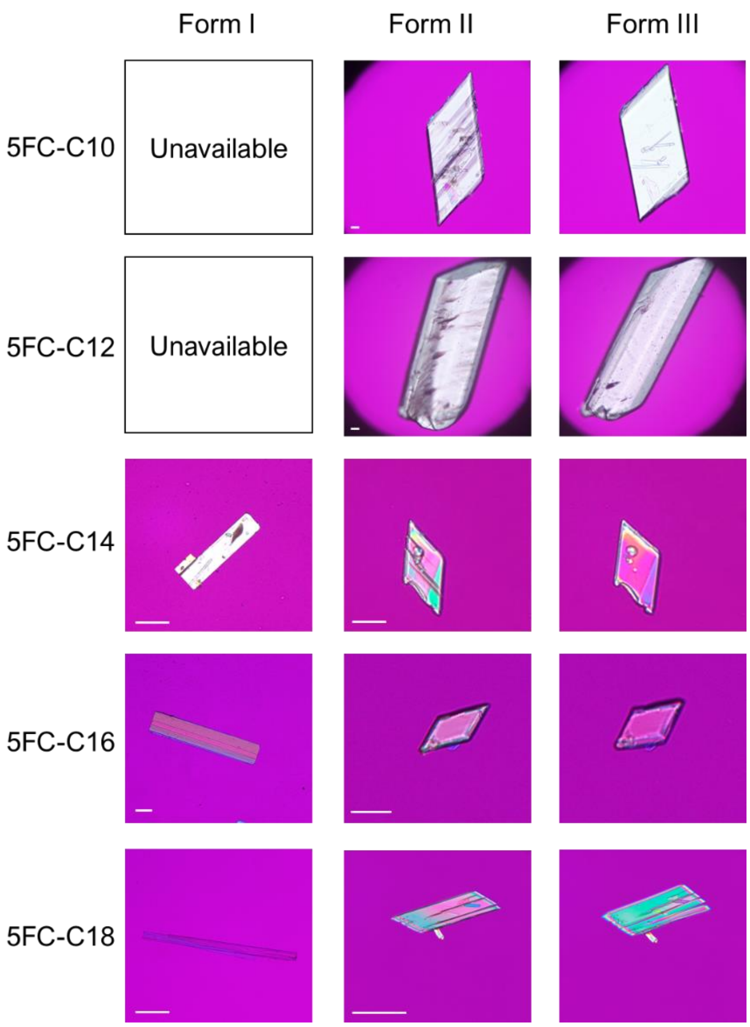

Figure S3. Crystal morphologies of 5FC-C2*n* polymorphs. The 5FC-C10 form I was not obtained in this study. Single crystals of 5FC-C12 form I were not isolated, although the powder form was successfully obtained using a slurry method in an ice water bath. Scale bar: 100 µm.

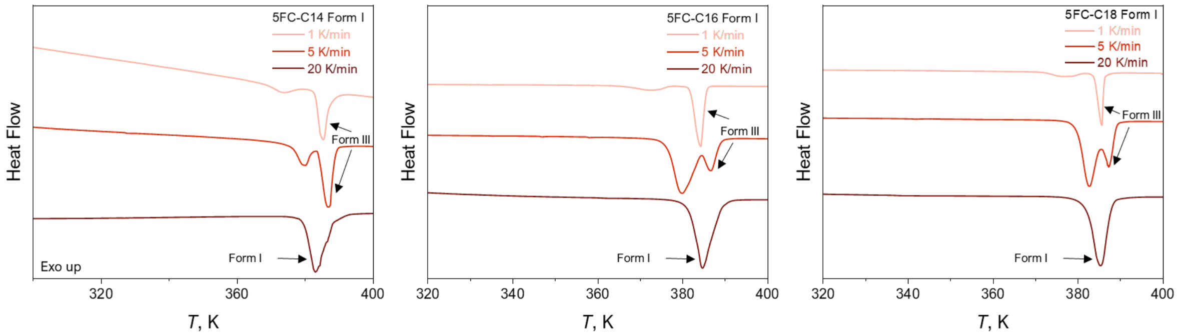

Figure S4. DSC profiles of form I with different heating rates in 5FC-C14, 5FC-C16 and 5FC-C18, respectively. The transformation from form I to form III was observed at slower heating rates.

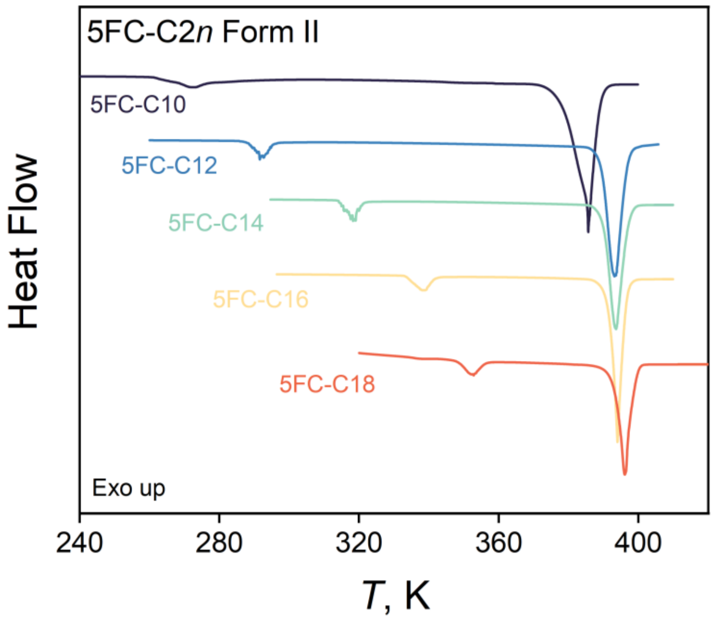

Figure S5. DSC profiles of powder samples of form II in the 5FC-C10/C12/C14/C16/C18 systems, measured at a heating rate of 20 K min^{-1}, show that form II undergoes a first-order endothermic transition (II to III) prior to melt. The transition temperature ($T_{t,\ II\text{-}III}$) varied significantly, spanning 250 to 350 K as the chain length increased from C10 to C18.

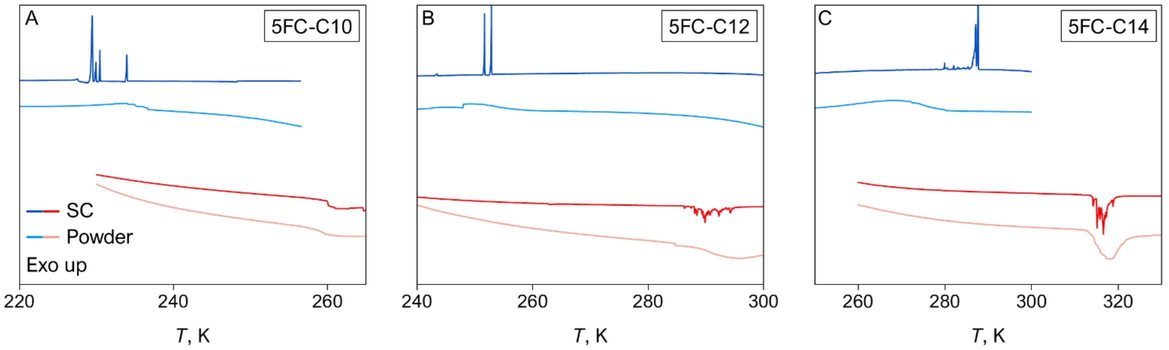

Figure S6. DSC profiles of single crystals and powders show that the phase transition (form II to form III) signals correlate with the crystal size. Due to the inability to prepare large-sized single crystals of 5FC-C16 and 5FC-C18, results are presented only for the 5FC-C10, 5FC-C12, and 5FC-C14 cocrystals.

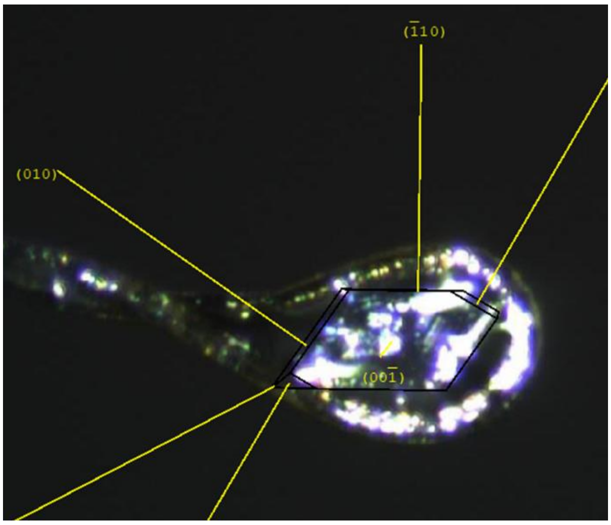

Figure S7. Macroscopic morphology of form II in 5FC-C14 with face index. The major surface is parallel to the $(00\bar{1})$ plane.

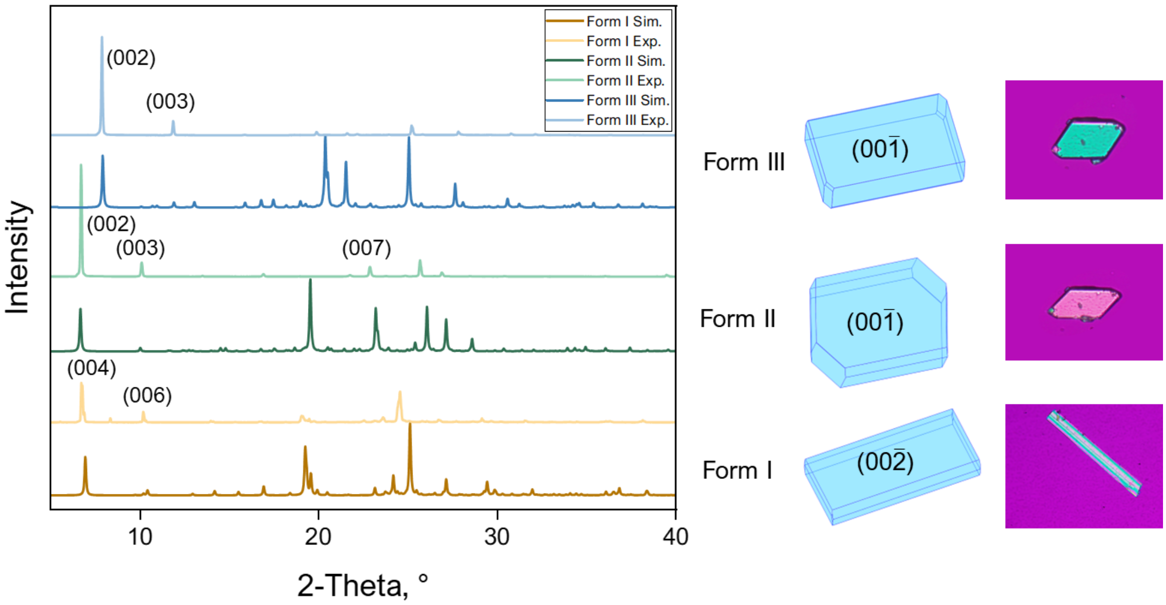

Figure S8. PXRD patterns and crystal morphologies of 5FC-C14 polymorphs. The simulated results correspond well with the experimental results. PXRD patterns reveal that the $\{00\bar{1}\}$ peaks shift to lower angles as the alkyl chain length increases in both forms II and III.

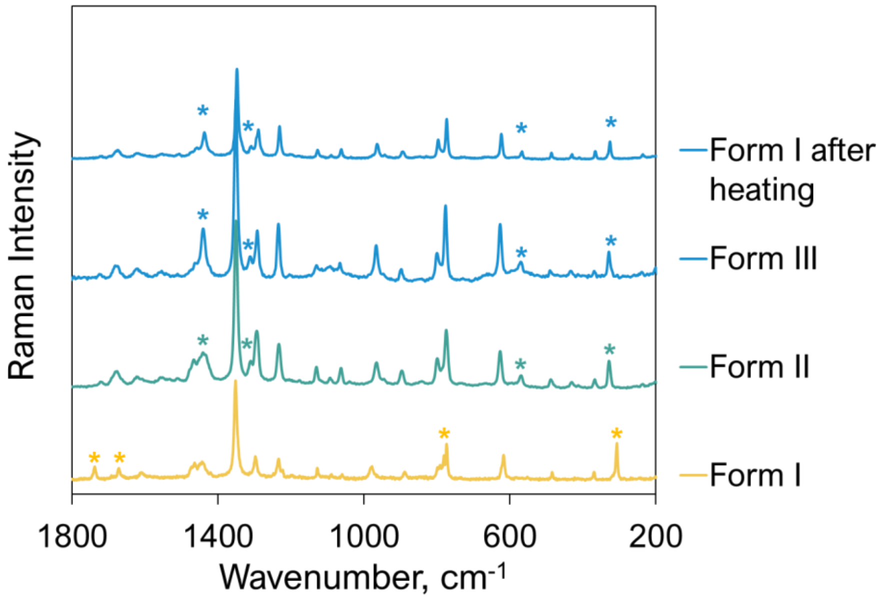

Figure S9. Raman spectra before and after the nucleation-to-growth phase transition in the 5FC-C14 system. Characteristic peaks of each form have been marked with asterisks. The results demonstrate that form I transforms into form III upon heating.

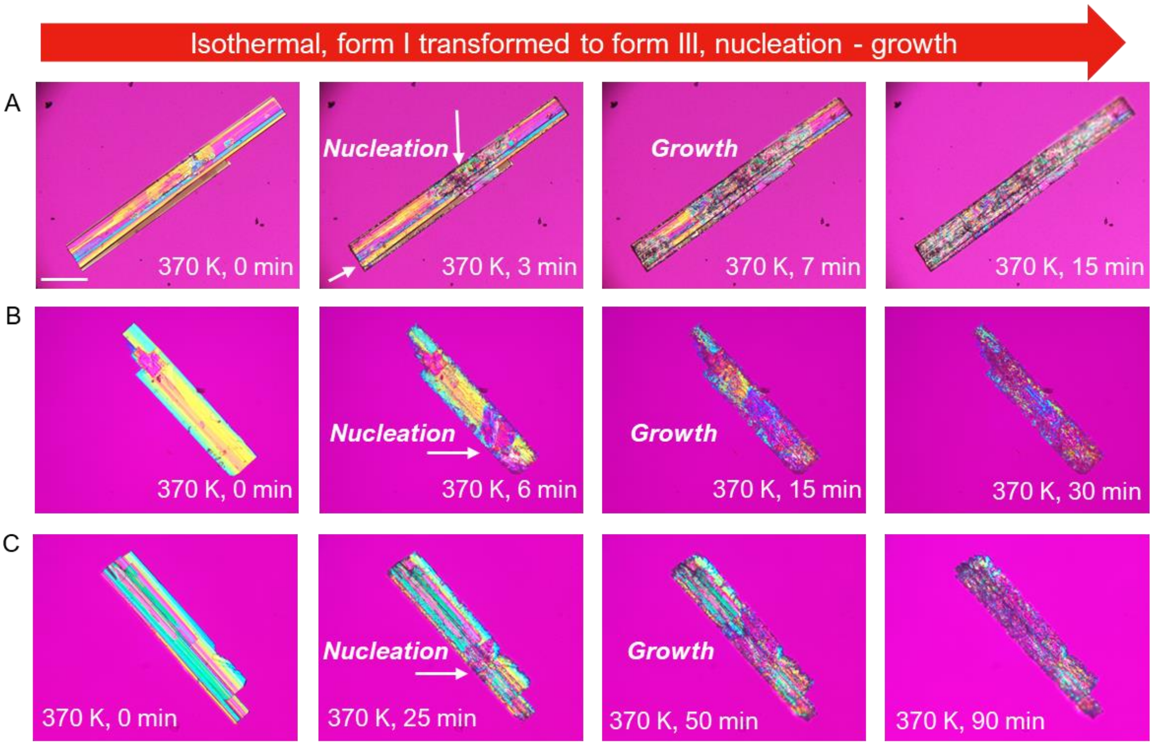

Figure S10. Nucleation-to-growth transition from form I to form III. Panels A to C show the transition for 5FC-C14, 5FC-C16, and 5FC-C18. Scale bar: 100 μm.

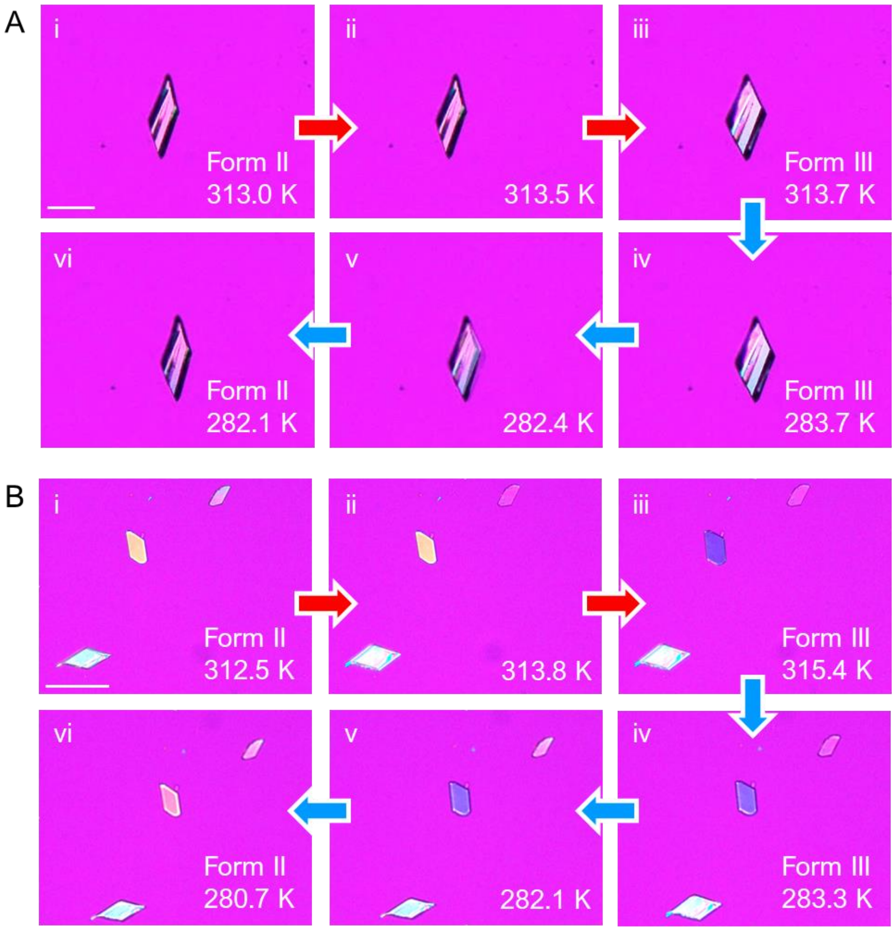

Figure S11. Reversible single-crystal-to-single-crystal (SCSC) phase transition of 5FC-C14 forms II and III (see Movies S1-S2). The crystals were immersed in silicon oil, and while the phase transition did not induce mechanical motions, but their morphology and interference color changed accordingly. Heating/cooling rate: 20 K/min. Scale bar: 100 μm.

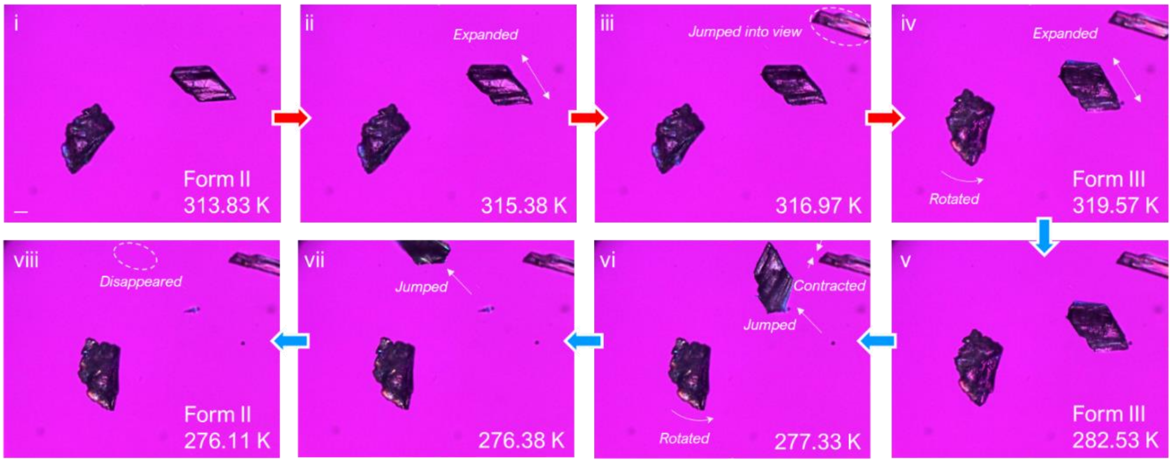

Figure S12. Thermosalient effects during the phase transition of 5FC-C14 from form II to form III (see Movie S8). Heating/cooling rate: 20 K/min. Scale bar: 100 μm.

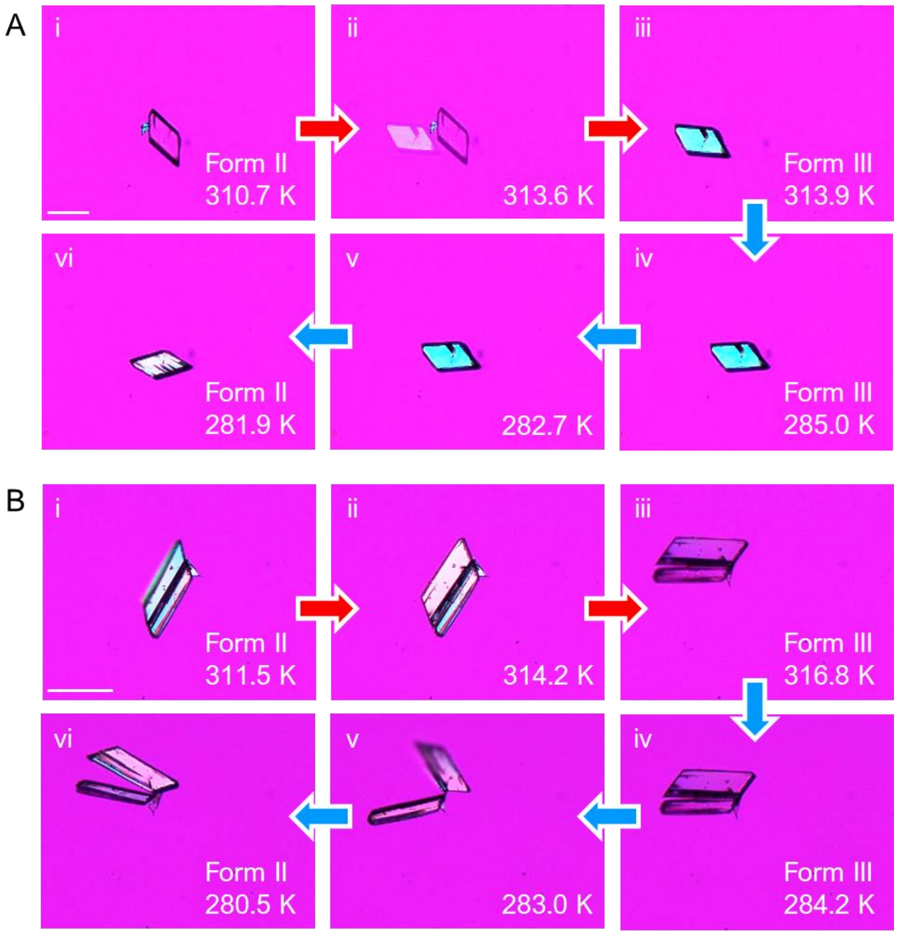

Figure S13. Thermosalient effects during the phase transition of 5FC-C14 from form II to form III (see Movies S9 and S10). Heating/cooling rate: 20 K/min. Scale bar: 100 µm.

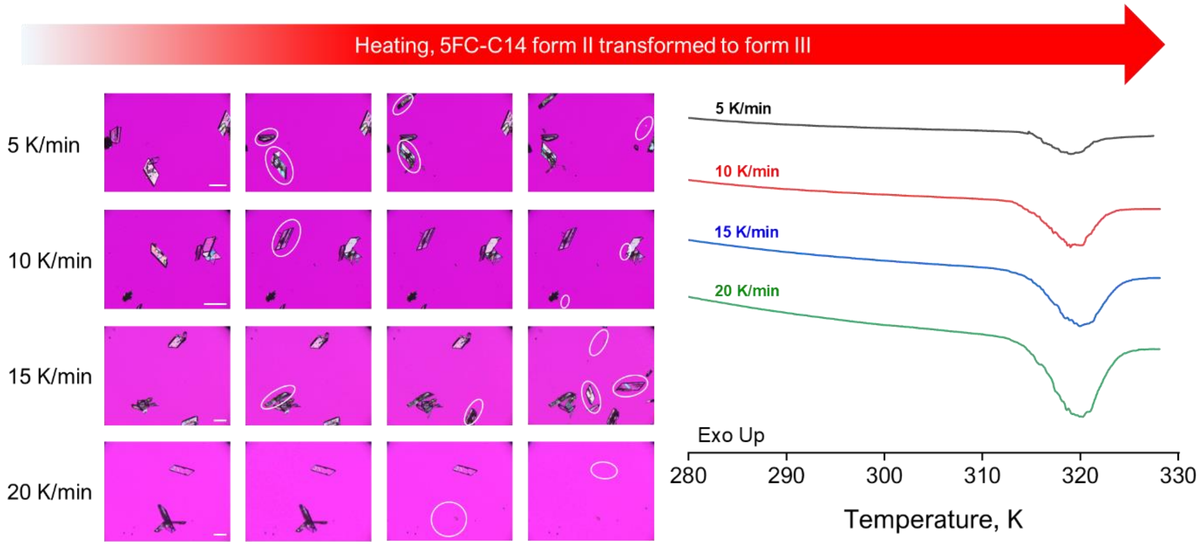

Figure S14. Thermosalient effects of 5FC-C14 cocrystals at different heating rates. The observed thermosalient behaviors are largely unaffected by variations in heating rates. Scale bar: 100 µm.

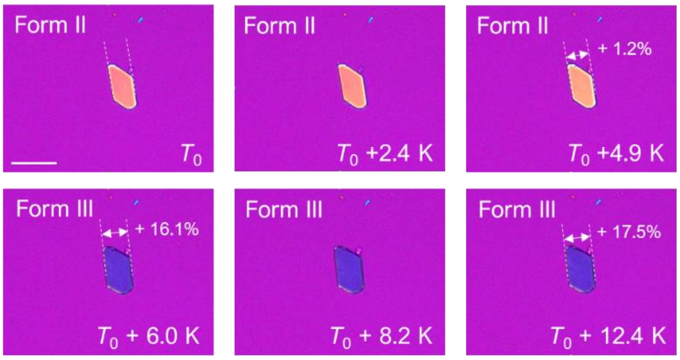

Figure S15. The expansion of a 5FC-C14 cocrystal during a heating process. The crystal expansion is measured relative to the initial reference temperature T_0. Scale bar: 100 μm.

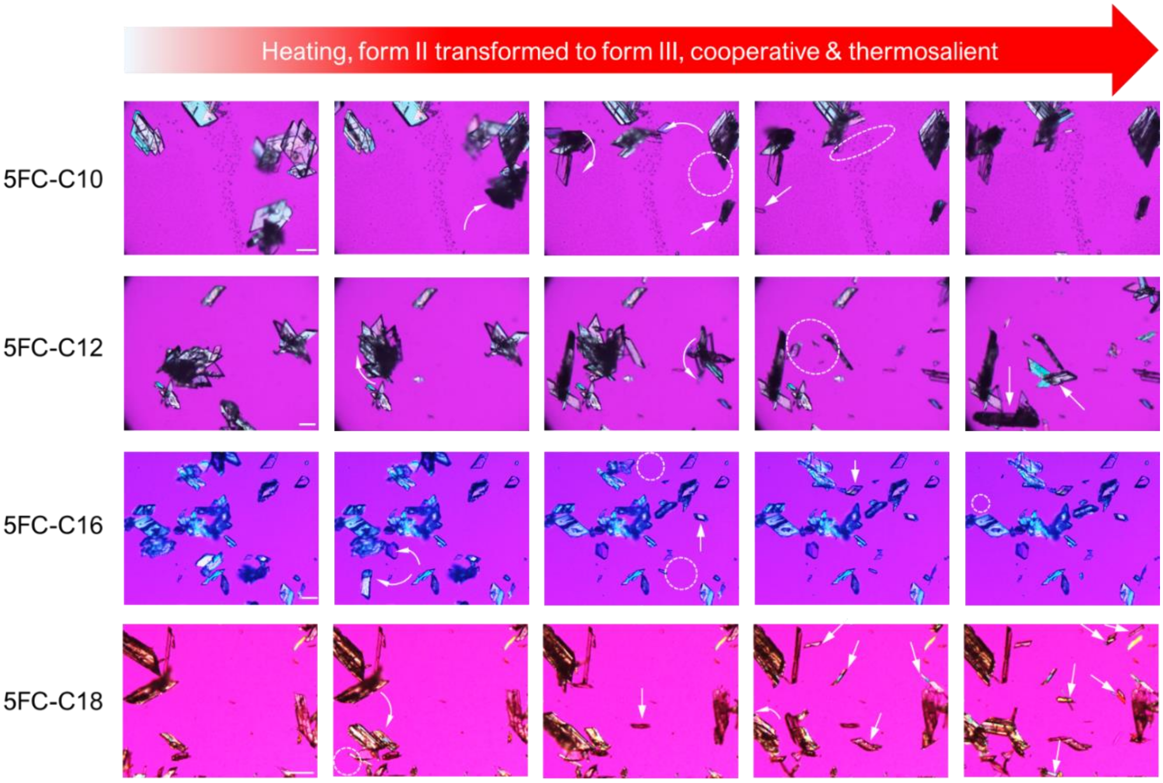

Figure S16. Thermosalient effects during the II-III phase transitions of 5FC-C2*n* cocrystals (see Movies S11-S14). Heating/cooling rate: 20 K/min. Scale bar: 100 μm.

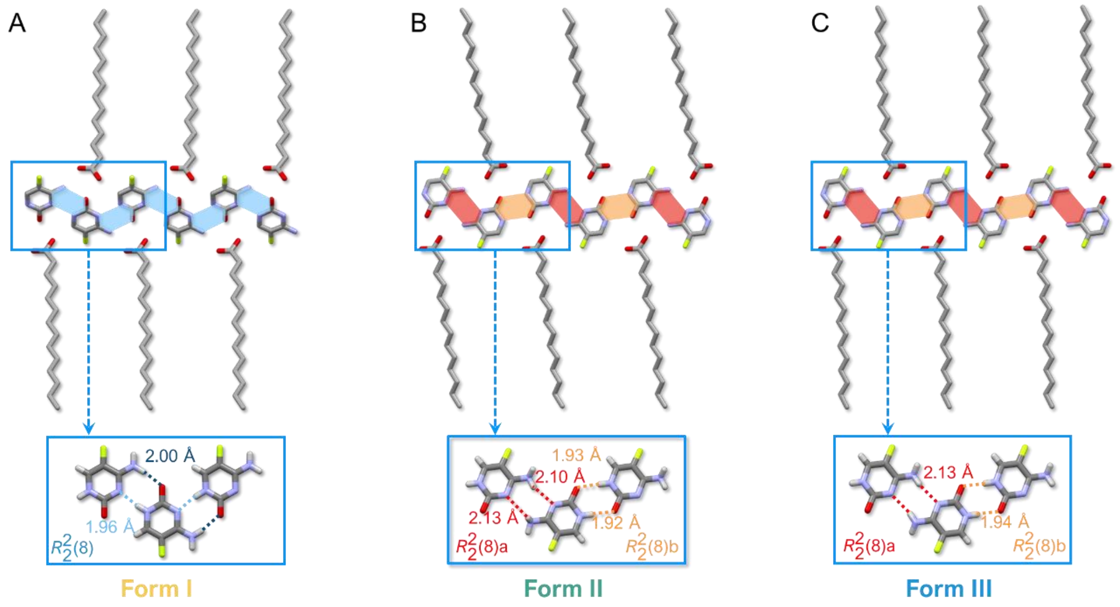

Figure S17. Structure motifs of 5FC-C14 polymorphs. (A) form I, (B) form II, (C) and form III. Hydrogen-bonded chains formed by 5FC in the three forms of 5FC-C14 cocrystal: (A) Ribbon motif 1 (RM1), graph set $R_2^2(8)$; (B) Ribbon motif 2 (RM2), graph set $R_2^2(8)$a, $R_2^2(8)$b.

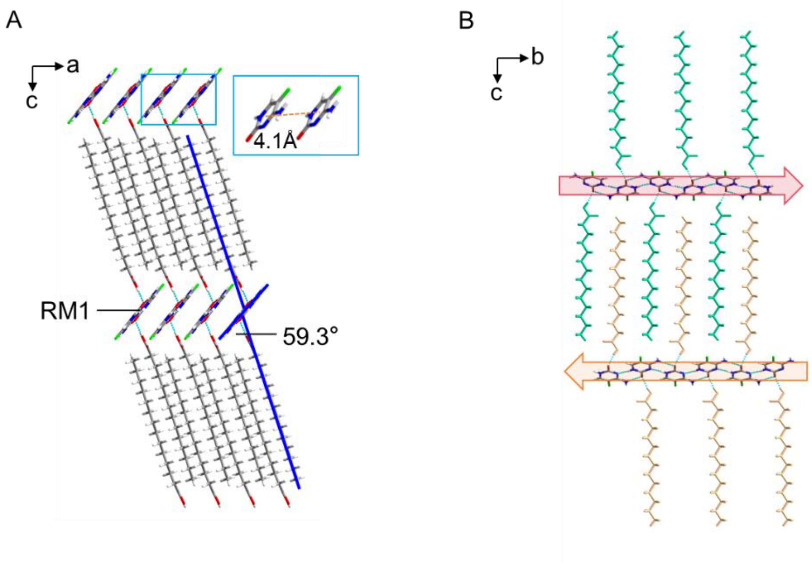

Figure S18. Crystal packing of 5FC-C14 form I viewed down the *b*-axis and *a*-axis, respectively. Form I crystallizes in a monoclinic system, featuring corrugated alkyl bilayers where the aromatic ribbons are tilted relative to the alkyl layers. These bilayers stack antiparallel along the *c*-axis, and the alkyl chains are offset along the *a*-axis.

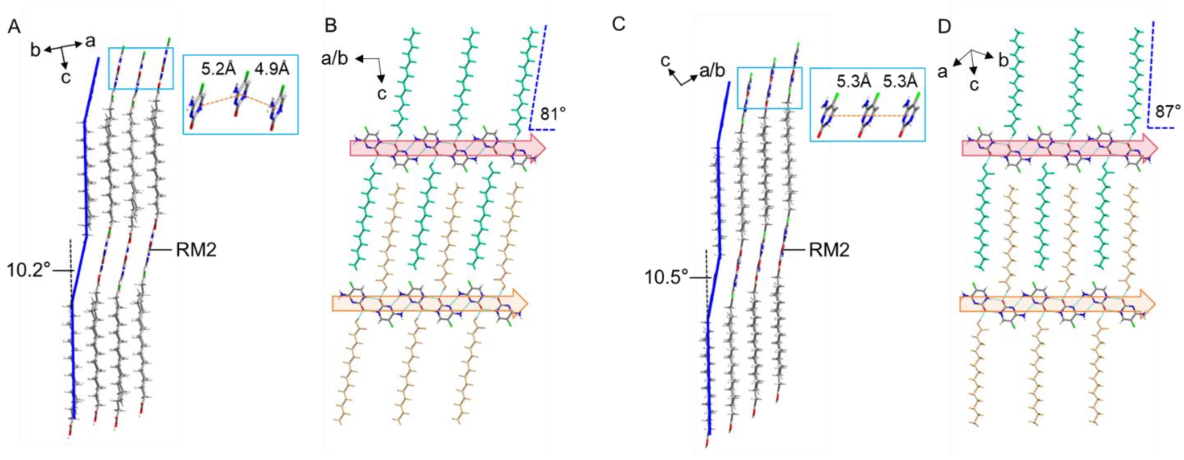

Figure S19. Crystal packing of 5FC-C14 form II (A, B) and form III (C, D). Centroid-centroid distances between the adjacent aromatic rings from the two ribbon motifs of the two forms are labeled in the enlarged image.

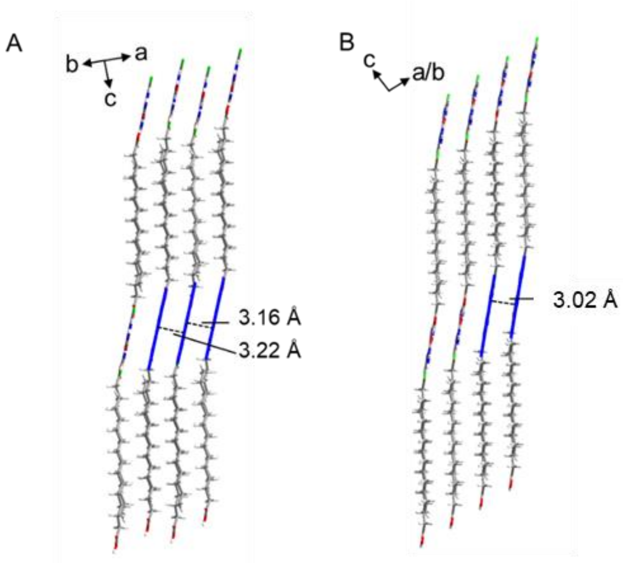

Figure S20. Interlayer distance in 5FC-C14 form II (A) and form III (B). Form II exhibits alternating interlayer distances of 3.22 and 3.16 Å, whereas form III shows a uniform interlayer distance of 3.02 Å.

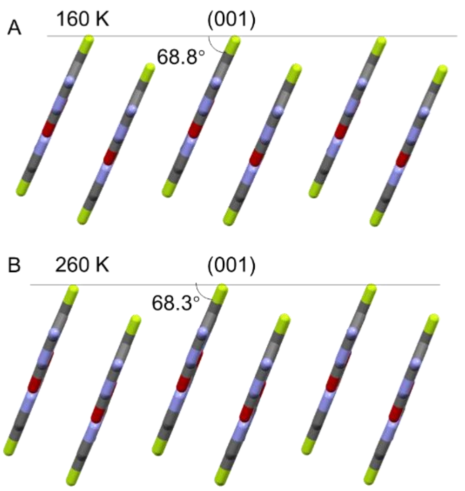

Figure S21. Tilt angle of the aromatic ring to the (001) plane in 5FC-C14 form II at 160 and 260 K.

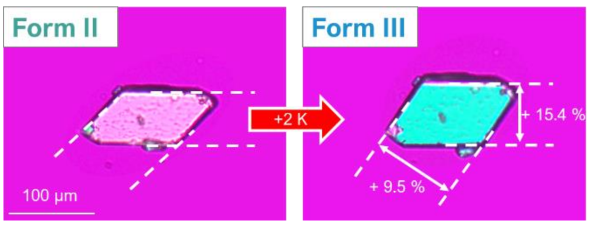

Figure S22. During phase transition (II to III), 5FC-C14 cocrystal expanded along the two sets of opposing edges measuring approximately 15.4% and 9.5%, respectively. These expansions correspond to the changes along the [1$\bar{1}$0] and [010] crystallographic directions in Form II.

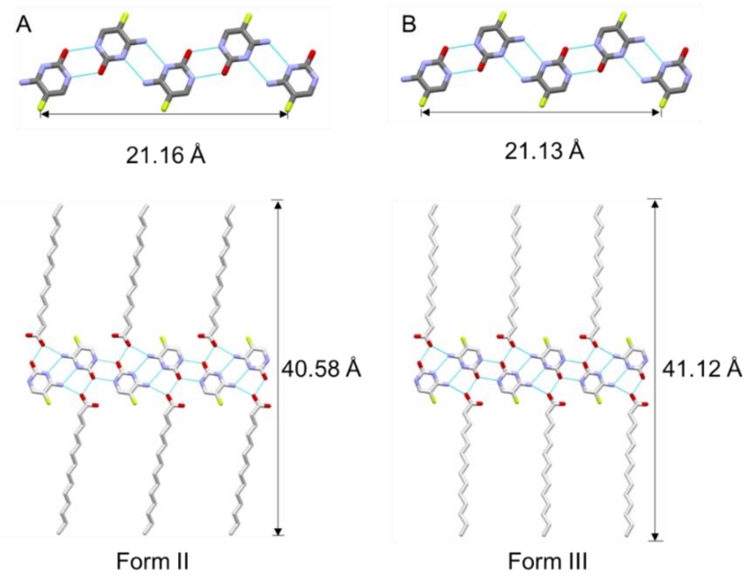

Figure S23. Length of the ribbon motif (forms II and III). The change along the 1D chain is negligible (-0.1%), while the change perpendicular the 1D chain within the layer is +1.33%.

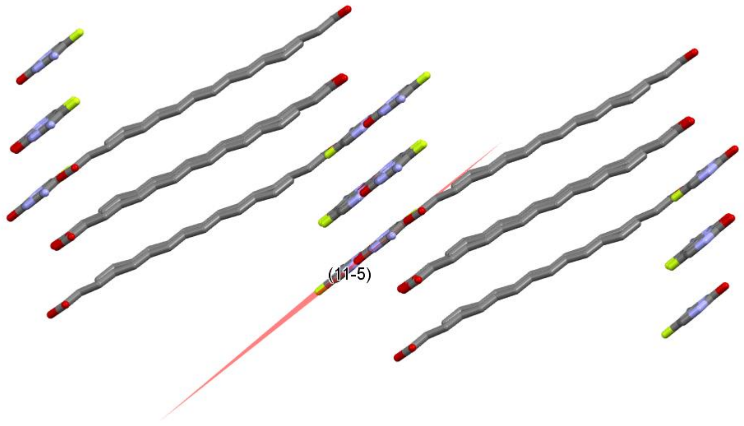

Figure S24. The layered structures are almost parallel to the (11$\bar{5}$) plane (5FC-C14 form III).

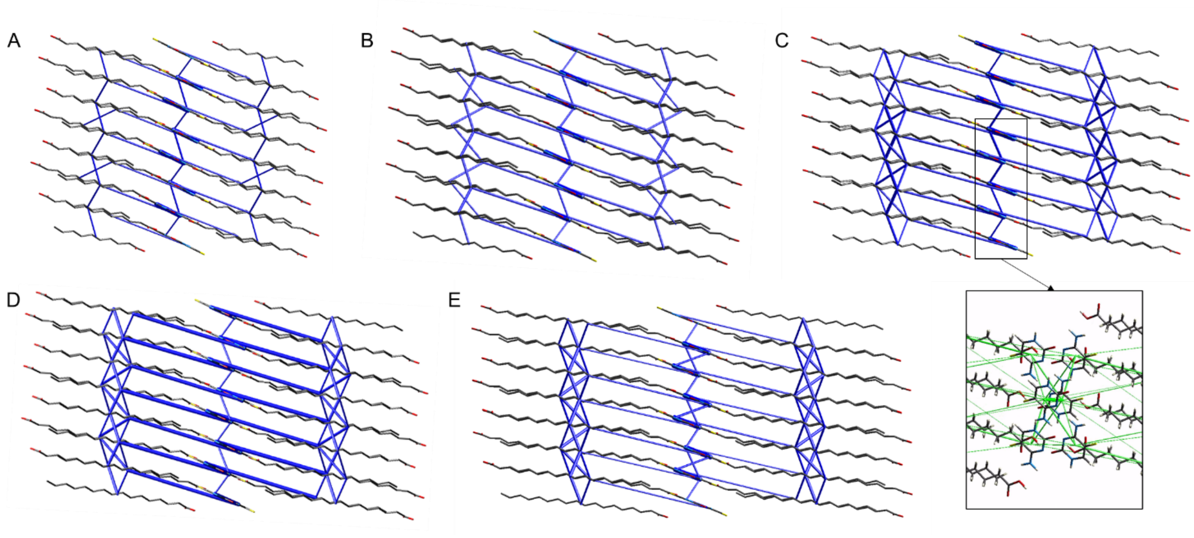

Figure S25. Energy framework of 5FC-C2*n* form II. (A) 5FC-C10, (B) 5FC-C12, (C) 5FC-C14, (D) 5FC-C16, and (E) 5FC-C18. The rod size in the energy frameworks is set to 30, and the cut-off total energy value is 30 kJ mol^{-1}.[1]

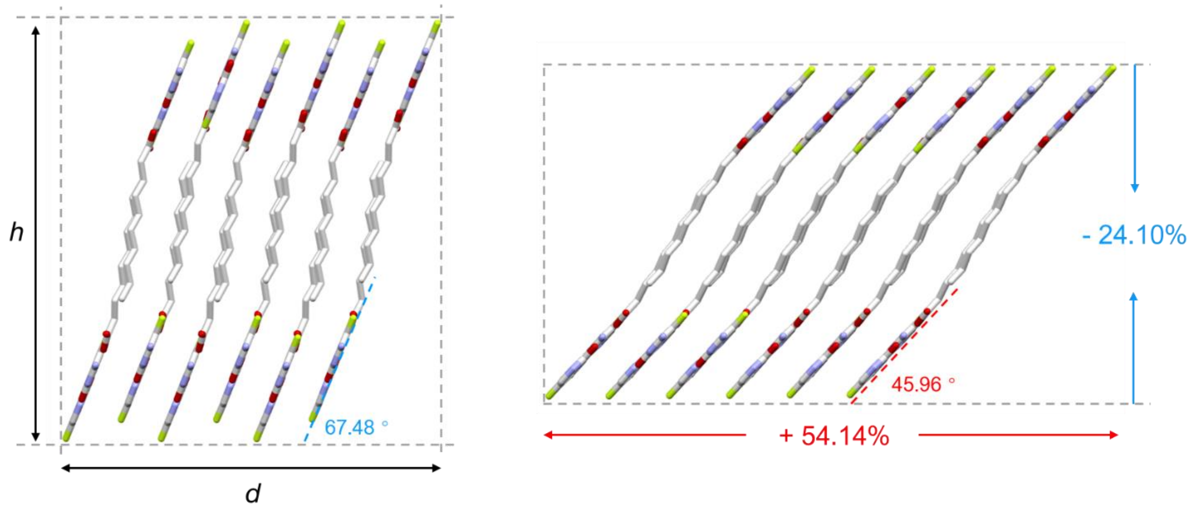

Figure S26. A perspective view of the layer packing mode of 5FC-C10 form II (left) at 213 K and form III (right) at 298 K. Upon heating from 213 to 298 K, the distance between the (001) plane decreases by 24.10%, while the direction along the layer stacking elongated by 54.14%.

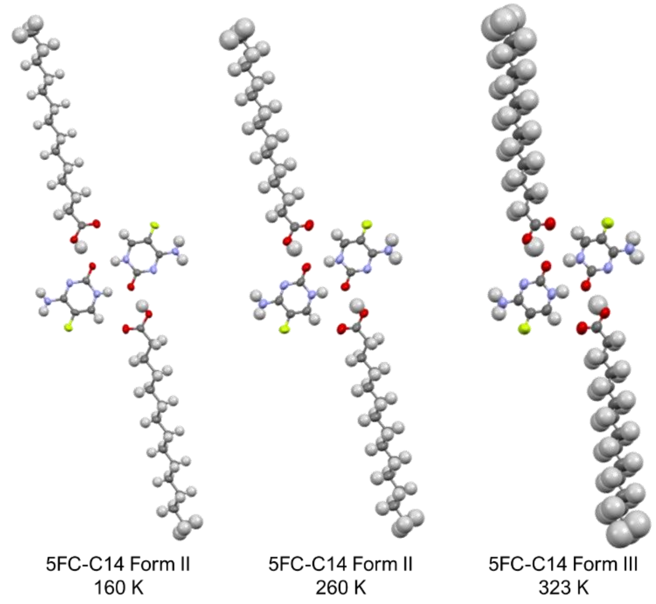

Figure S27. The thermal ellipsoid plots of 5FC-C14 form II at 160 and 260 K. All the structures are presented with 80% thermal ellipsoids.

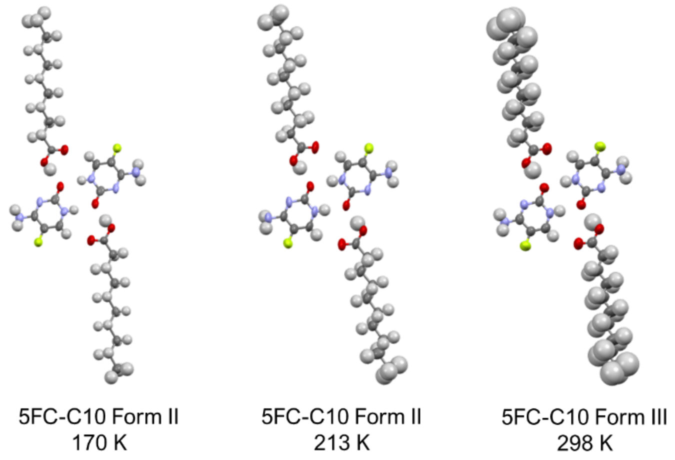

Figure S28. The thermal ellipsoid plots of 5FC-C10 form II at 170 and 213 K. All the structures are presented with 80% thermal ellipsoids.

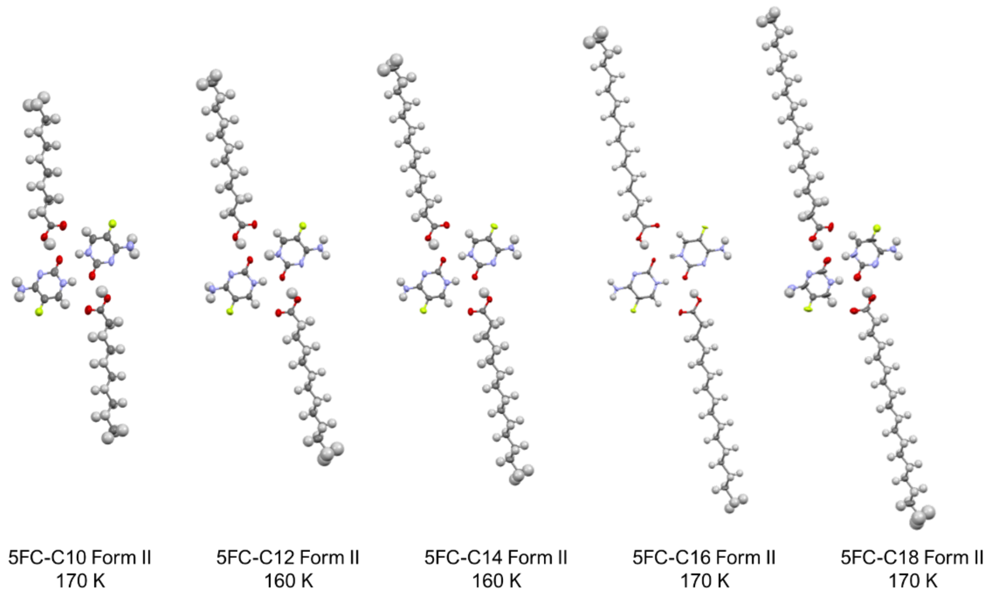

Figure S29. The thermal ellipsoid plots of 5FC-C2*n* form II. All the structures are presented with 80% thermal ellipsoids.

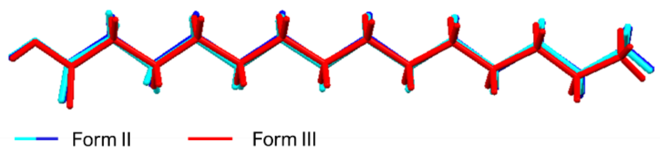

Figure S30. Conformation superposition of fatty acid in 5FC-C14 form II and III. The blue and red color schemes correspond to the molecular conformations of fatty acids in form II and form III, respectively. Specifically, the cyan and blue regions represent two distinct fatty acid molecules within the asymmetric unit of form II.

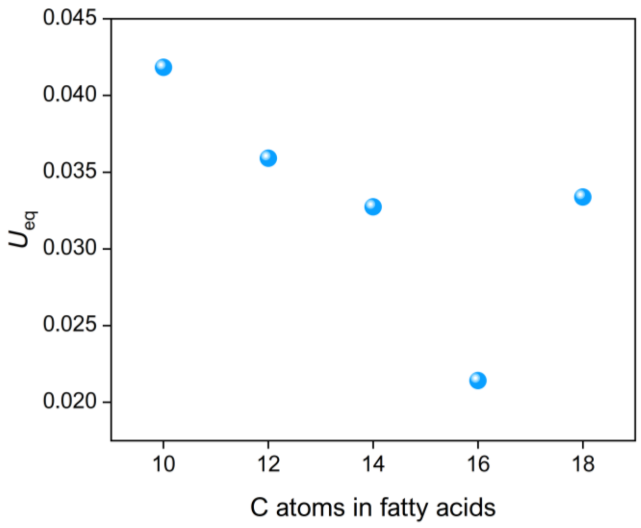

Figure S31. The average atomic displacement parameters (U_{eq}) as the function of the alkyl chains in 5FC-C2*n* form II. There is no significant correlation between molecular mobility and the length of the alkyl chain.

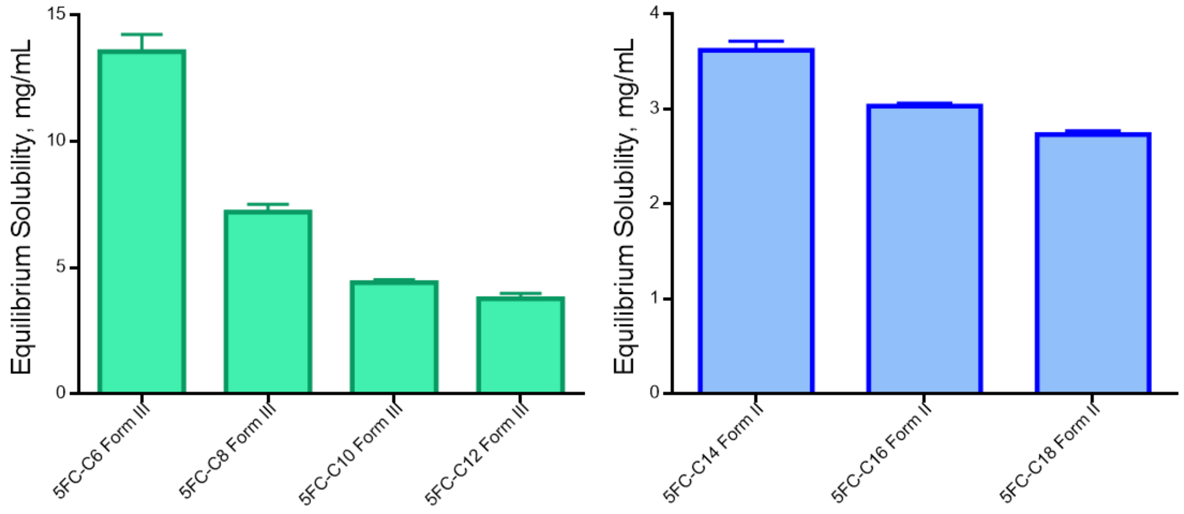

Figure S32. Dissolution properties of 5FC-C2*n* (A) form II and (B) form III (pH = 6.8). Solubility in near-neutral environments decreases with increasing alkyl chain length.

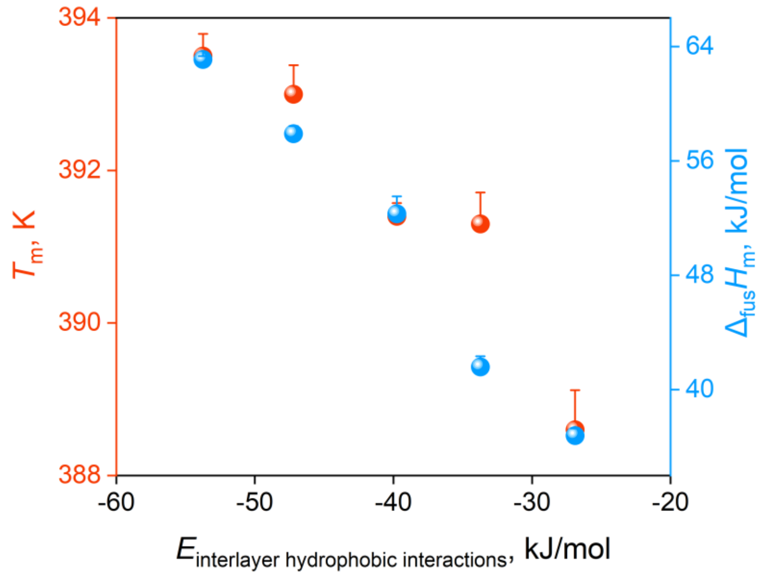

Figure S33. Correlation between the thermodynamic parameters (T_m and $\Delta_{fus}H_m$) and the $E_{interlayer}$ of 5FC-C2*n* cocrystals.

Table S1. The melting temperature (T_m) and $\Delta_{fus}H_m$ of 5FC-C2*n* form I and form III.

Form	5FC-C14 I	5FC-C14 III	5FC-C16 I	5FC-C16 III	5FC-C18 I	5FC-C18 III
T_m (K)	379.39 (17)	391.40 (8)	381.98 (5)	393.03 (4)	382.24 (8)	393.47 (9)
$\Delta_{fus}H_m$ (kJ/mol)	67.24 (45)	52.33 (23)	72.56 (13)	57.94 (25)	79.94 (18)	63.15 (19)
$T_{t,\ \mathrm{I\ to\ III}}$	342.49		343.66		345.19	

Table S2. Crystallographic data of 5FC-C2*n* form II.

	5FC-C10 form II	5FC-C12 form II	5FC-C14 form II	5FC-C14 form II	5FC-C16 form II	5FC-C18 form II
Empirical formula	$C_{14}H_{24}FN_3O_3$	$C_{16}H_{28}FN_3O_3$	$C_{18}H_{32}FN_3O_3$	$C_{18}H_{32}FN_3O_3$	$C_{20}H_{36}FN_3O_3$	$C_{22}H_{40}FN_3O_3$
CCDC number	2410439	2410434	2410438	2410435	2410437	2410436
Formula weight	301.36	329.41	357.46	357.46	385.52	413.57
Temperature/K	213	160	160	260	170	170
Crystal system	Triclinic	Triclinic	Triclinic	Triclinic	Triclinic	Triclinic
Space group	*P*-1	*P*-1	*P*-1	*P*-1	*P*-1	*P*-1
a/Å	7.972(7)	7.913(2)	7.907(3)	8.0444(5)	7.862(17)	7.893(3)
b/Å	9.645(8)	9.633(3)	9.685(3)	9.7483(6)	9.654(18)	9.678(3)
c/Å	21.980(3)	24.357(6)	26.781(9)	26.7008(7)	29.033(5)	31.459(10)
α/°	100.32(4)	97.85(9)	95.894(9)	96.065(4)	94.30(7)	92.81(10)
β/°	94.33(5)	94.13(9)	93.823(9)	93.929(4)	93.33(8)	92.94(13)
γ/°	107.04(2)	106.48(10)	106.507(10)	107.753(5)	106.54(8)	106.83(12)
Volume/Å^3	1575(3)	1751.7(8)	1946.0(11)	1971.73(19)	2099.3(7)	2291.9(13)
Z	4	4	4	4	4	4
ρ_{calc} g/cm^3	1.271	1.249	1.220	1.204	1.22	1.199
μ/mm^{-1}	0.097	0.093	0.089	0.724	0.088	0.085
F(000)	648	712	776	776	840	904

Table S3. Crystallographic data of 5FC-C2*n* form I and form III.

	5FC-C6 form III	5FC-C10 form III	5FC-C14 form I	5FC-C14 form III
Empirical formula	$C_{10}H_{16}FN_3O_3$	$C_{14}H_{24}FN_3O_3$	$C_{18}H_{32}FN_3O_3$	$C_{18}H_{32}FN_3O_3$
CCDC number	2413835	2410432	2410433	2410440
Formula weight	245.26	301.36	357.46	357.46
Temperature/K	170	298	163	323
Crystal system	Triclinic	Triclinic	Monoclinic	Triclinic
Space group	*P*-1	*P*-1	$P2_1/n$	*P*-1
a/Å	5.2475(4)	5.2859(17)	4.1043(8)	5.2908(2)
b/Å	8.7634(7)	8.793(3)	9.213(2)	8.7801(3)
c/Å	13.6669(14)	18.052(6)	50.880(10)	22.2857(6)
α/°	77.297(4)	83.496(9)	90	91.695(3)
β/°	89.727(3)	83.659(10)	91.41(6)	93.015(3)
γ/°	84.927(3)	85.821(10)	90	94.050(3)
Volume/Å^3	610.63(9)	826.9(4)	1923.4(7)	1030.66(6)
Z	2	2	4	2
ρ_{calc} g/cm^3	1.334	1.210	1.234	1.152
μ/mm^{-1}	0.109	0.093	0.09	0.692
F(000)	260	324	776	388

The crystal structure of 5FC-C8 form III has been reported by M. S. Souza et al., and the CCDC number is 1817635.[2]

Table S4. The average atomic displacement parameters (U_{eq}) of the alkyl chains in 5FC-C2*n* form II.

	5FC-C10	5FC-C12	5FC-C14	5FC-C16	5FC-C18
U_{eq}	0.04184	0.03592	0.03275	0.02142	0.03339

References

[1] P. R. Spackman, M. J. Turner, J. J. McKinnon, S. K. Wolff, D. J. Grimwood, D. Jayatilaka, M. A. Spackman, *J.Appl. Crystallogr.* **2021**, *54*, 1006-1011.

[2] M. S. Souza, L. F. Diniz, L. Vogt, P. S. Carvalho, R. F. D'vries, J. Ellena, *New J. Chem.* **2018**, *42*, 14994-15005.
